# Coordinatively Unsaturated Hf-MOF-808 Prepared via Hydrothermal Synthesis as a Bifunctional Catalyst for the Tandem *N*-Alkylation of Amines with Benzyl Alcohol

**DOI:** 10.1021/acssuschemeng.1c04903

**Published:** 2021-11-17

**Authors:** Benjamin Bohigues, Sergio Rojas-Buzo, Manuel Moliner, Avelino Corma

**Affiliations:** Instituto de Tecnología Química, Universitat Politècnica de València - Consejo Superior de Investigaciones Científicas, Av. de los Naranjos, s/n, 46022 Valencia, Spain

**Keywords:** MOF-808, hydrothermal synthesis, tandem reaction, *N*-alkylation, borrowing hydrogen

## Abstract

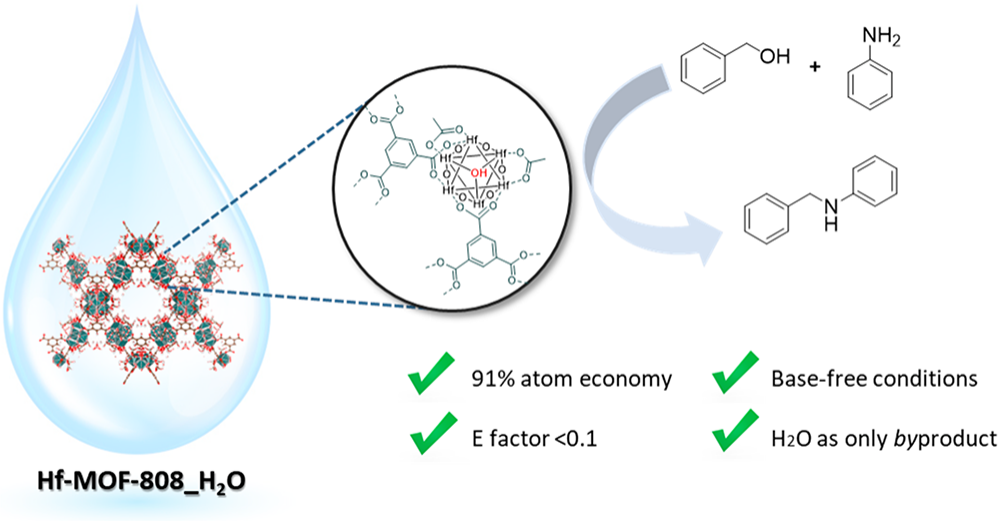

The modulated hydrothermal (MHT) synthesis of an active and selective Hf-MOF-808 material for the *N*-alkylation reaction of aniline with benzyl alcohol under base-free mild reaction conditions is reported. Through kinetic experiments and isotopically labeled NMR spectroscopy studies, we have demonstrated that the reaction mechanism occurs via borrowing hydrogen (BH) pathway, in which the alcohol dehydrogenation is the limiting step. The high concentration of defective −OH groups generated on the metallic nodes through MHT synthesis enhances the alcohol activation, while the unsaturated Hf^4+^, which acts as a Lewis acid site, is able to borrow the hydrogen from the methylene position of benzyl alcohol. This fact makes this material at least 14 times more active for the *N*-alkylation reaction than the material obtained via solvothermal synthesis. The methodology described in this work could be applied to a wide range of aniline and benzyl alcohol derivates, showing in all cases high selectivity toward the corresponding *N*-benzylaniline product. Finally, Hf-MOF-808, which acts as a true heterogeneous catalyst, can be reused in at least four consecutive runs without any activity loss.

## Abstract 2

Sustainable hydrothermal synthesis of Hf-MOF-808 considerably improves its catalytic activity for the tandem *N*-alkylation reaction avoiding the use of external H_2_ and homogeneous bases.

## Introduction

Industry is more than ever directed toward the development of efficient synthetic routes that minimize chemical waste, the use of hazardous raw sources, and/or the number of required steps. To this respect process intensification by cascade reactions that imply multiple consecutive transformations in the same reaction system is attracting a lot of attention in the field of catalysis.^1−4^ These types of intensive processes present several advantages, including separation and purification intermediate steps that may also result in kinetic and production advantages.^4^

Among different chemical reactions, those comprising hydrogen auto-transfer mechanisms (borrowing hydrogen, BH)^5^ stand out as a powerful approach for the *one-pot* formation of C–C^6,7^ or C–N^8−11^ bonds without requiring intermediate purification steps. The direct *N*-alkylation reaction of amines with alcohols offers an attractive pathway for the synthesis of secondary amines with high applicability in the pharmaceutical industry, materials science, agrochemistry, and biological systems. It is worth noting that the synthesis of secondary amines through the direct *N*-alkylation reaction of amines with alcohols shows outstanding benefits: (a) no hydrogen acceptor or oxidant is required, (b) water is only generated as a byproduct, a fact that improves the sustainability of the process compared to the generation of inorganic salts produced in classical amination reactions (i.e., from alkyl or aryl halides),^8^ (c) high atomic efficiency can be achieved, (d) alcohols are employed as both alkylating agents and hydrogen sources, being then cheaper, less flammable, and better manipulated compared to H_2_, and (e) side reactions are minimized (i.e., aldol condensation between alcohols and/or over alkylation reaction).

**Scheme 1:**
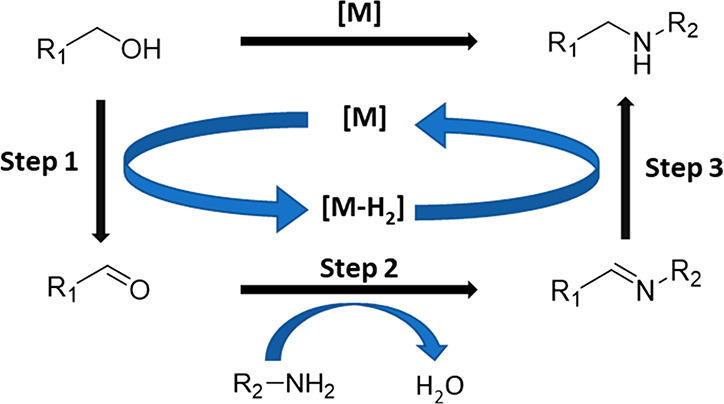
Hydrogen Auto-transfer Mechanism for the *N*-Alkylation Reaction of Amines with Alcohols Mediated by Transition Metal-Based Materials

The *N*-alkylation of amines using alcohols has been mostly associated in the literature with a hydride-capture mechanism by metal-containing catalysts, where the general mechanism consists of three well-defined stages:^5^ (1) alcohol dehydrogenation and hydride capture by the metal-containing catalyst, (2) condensation between the carbonyl group generated and the amine giving rise to an imine, and (3) imine hydrogenation by the metal hydride produced in the initial dehydrogenation stage (Scheme 1). However, it is worth noting that recent descriptions have also reported that the stripped hydrogen during the alcohol dehydrogenation can be stored in the form of hydrazo in the ligand backbone without metal–hydride formation.^12^

Numerous studies have shown that noble and non-noble metal complexes, mainly based on Ru,^13,14^ Ir,^15^ Ni,^12,16,17^ Mn,^18^ Co,^19,20^ Cu,^21^ and Fe,^22^ combined with co-catalysts, such as bases and stabilizing ligands to undergo the alcohol deprotonation, are efficient multifunctional catalytic systems for the *N*-alkylation reaction. However, these homogeneous catalytic systems offer some drawbacks associated with their high cost and/or their limited recovery/regeneration. To circumvent these limitations, different heterogeneous catalysts based on supported transition metals have been described in the literature,^9,10,23^ but most of these solids still present some disadvantages, including uncertain stability/recyclability and/or the use of homogeneous bases.

Metal–organic frameworks (MOFs) are a very versatile family of microporous materials formed by inorganic metallic nodes connected through organic ligands,^24^ where the nature of the metallic cluster and/or the organic ligand can be easily tuned by direct synthesis or postsynthetic treatments, offering unique capabilities as heterogeneous catalysts.^25,26^ Indeed, MOFs can be considered as interesting catalysts for tandem reactions since these materials allow the simultaneous introduction of different active sites.^27,28^ Despite the high-tunability of MOFs, only two examples of MOF-based materials have been reported as catalysts for the *N*-alkylation reaction.^27,29^ The first description was a heterogenized Ir complex on the organic ligand of the Zr-UiO-66-NH_2_ material, which allows performing the *N*-alkylation reaction under solvent and base-free conditions.^27^ This methodology describes excellent results for aliphatic reagents, but the overall efficiency of the process is considerably reduced when using aromatic substrates, such as benzyl alcohol and aniline. Moreover, the initial catalytic activity decreases along the different catalytic cycles, indicating a limited stability for the heterogenized Ir complex. More recently, a MOF-type material based on the MIL-125-NH_2_ structure has been described for this reaction.^29^ In this catalytic system, the unsaturated Ti^4+^ sites of the metallic nodes and the O-atoms of Ti–O clusters would provide vicinal Lewis acid and Lewis basic sites, respectively, facilitating alcohol adsorption and its subsequent dehydrogenation reaction (borrowing hydrogen). The selectivity toward the corresponding *N*-benzylaniline product obtained in this work is higher than 98%, but this catalytic system requires almost 15 h and 160 °C to achieve complete conversion values.

Recently, we have demonstrated that transition metal-containing beta zeolites, particularly Zr- and Hf-beta, show excellent activity, selectivity, and stability for the *N*-alkylation reaction of aniline with benzyl alcohol under base-free conditions.^30^ These previous results suggest that the Lewis acid strength offered by the isolated transition metal sites in the zeolite framework would play an important role on the alcohol dehydrogenation (step 1 in Scheme 1).^30,31^ Thus, the preparation of a MOF-type catalyst including tetravalent species other than Ti with enhanced Lewis acidity, such as Zr and Hf,^31^ can give rise to a faster metal hydride formation and, therefore, a remarkable activity improvement compared to Ti-containing MIL-125-NH_2_.^29^ It is worth noting that, in the last years, Zr- and Hf-MOFs have shown exceptional thermal, mechanical, and chemical stabilities,^32−34^ allowing their application as active and stable catalysts for a wide range of chemical processes.^35−40^

The control of the amount of coordinatively unsaturated open metal sites in MOFs has been demonstrated crucial in rationalizing the nature of the catalytically active sites, allowing fine-tuning the Lewis/Brønsted acid properties of the resultant MOFs.^41,42^ Different strategies have been described in the literature to adequately generate these defective sites, such as the use of modulators (i.e., monocarboxylic or inorganic acids) during the solvothermal synthesis,^43,44^ or postsynthetic modifications through acid/base treatments.^45^ In fact, the amount of defective −OH groups on the metallic cluster of Hf-containing MOFs can be modulated by carrying out their syntheses via solvothermal or hydrothermal conditions.^46^

Herein, we propose the use of Zr- and Hf-containing MOF materials as efficient and active catalysts for the synthesis of secondary amines via *N*-alkylation reaction between anilines and benzyl alcohols that does not require the use of an additional base and/or external H_2_, as required for instance when synthesizing secondary amines by the traditional condensation between amines and aldehydes. The metal-containing MOFs prepared via modulated hydrothermal synthesis conditions show a remarkably higher catalytic activity compared to the metal-containing MOFs prepared via modulated solvothermal synthesis, suggesting that a larger amount of −OH sites within the metal nodes substantially facilitates the *N*-alkylation reaction. Kinetic and NMR spectroscopy studies clearly reveal that the reaction occurs via a borrowing hydrogen pathway, in which the alcohol dehydrogenation is the limiting step. Finally, the Hf-MOF-808 catalyst has demonstrated good stability in this transformation, since it can be reused at least 4 times without observing any catalyst deactivation. The catalytic system can be expanded to the use of a large number of aromatic amines and benzyl alcohols.

## Experimental Section

### Metal–Organic Framework Synthetic Procedures

#### Modulated Hydrothermal Synthesis of M-MOF-808_H_2_O

This synthesis has been carried out following a previously reported recipe:^47^ MClO_2_·8H_2_O (M = Hf/Zr) (3.60 mmol) and H_3_BTC (1.20 mmol, 252.17 mg) were dissolved in a mixture of H_2_O/acetic acid (1:1, v/v, 20 mL). The resulting solution was refluxed at 100 °C under magnetic stirring for 37 h. The white solid precipitate was washed three times with H_2_O, methanol, and, finally, acetone. The as-obtained material, denoted as M-MOF-808_H_2_O, was activated at 100 °C for 2 h (1.21 g of Hf-MOF-808_H_2_O, 3.06 mmol of Hf, 85% yield referred to HfOCl_2_·8H_2_O; 0.97 g of Zr-MOF-808_H_2_O, 2.81 mmol of Zr, 78% yield referred to as ZrOCl_2_·8H_2_O).

#### Modulated Solvothermal Synthesis of Hf-MOF-808_DMF

This material has been synthesized according to a previously reported procedure:^48^ H_3_BTC (1,3,5-benzenetricarboxylic acid) (3.00 mmol, 0.63 g) and HfCl_4_ (3.00 mmol, 0.96 g) were dissolved in a mixture of DMF/formic acid (1:1, v/v, 90 mL) at reflux (100 °C) under magnetic stirring for 2 weeks. The precipitate was washed three times with DMF and acetone. The as-obtained white solid, denoted as Hf-MOF-808_DMF, was activated at 100 °C for 2 h (0.73 g of Hf-MOF-808_DMF, 2.31 mmol of Hf, 77% yield referred to as HfCl_4_).

#### Modulated Hydrothermal Synthesis of Hf-UiO-NH_2__H_2_O

This material has been synthesized according to a previously reported procedure:^49^ HfClO_2_·8H_2_O (5.20 mmol, 2.13 g) and 2-aminoterephthalic acid (5.00 mmol, 0.91 g) were mixed with H_2_O and acetic acid solution (30 and 20 mL, respectively). The resulting mixture was kept stirred and refluxed at 120 °C for 20 h. The yellow solid precipitate was washed alternatively three times with water, methanol, and, finally, acetone. The as-obtained material, denoted as Hf-UiO-NH_2__H_2_O, was activated at 100 °C for 2 h (2.29 g of Hf-UiO-NH_2__H_2_O, 4.78 mmol of Hf, 92% referred to as HfOCl_2_·8H_2_O).

### Characterization

#### General Characterization Techniques

Powder X-ray diffraction (PXRD) measurements were performed using a Panalytical CubiX diffractometer operating at 40 kV and 35 mA and using Cu Kα radiation (λ = 0.1542 nm).

Chemical analyses were carried out in a Varian 715-ES ICP-Optical Emission spectrometer, after solid dissolution in H_2_SO_4_/H_2_O_2_ aqueous solution. Elemental analyses were performed by combustion analysis using a Eurovector EA 3000 CHNS analyzer with sulfanilamide as the reference.

The sample morphology was studied by field emission scanning electron microscopy (FESEM) using a ZEISS Ultra-55 microscope.

The adsorption and desorption curve of N_2_ was measured at −196 °C in an ASAP2420 Micromeritics device. The specific surface areas were calculated by the Brunauer–Emmett–Teller (BET) method following Rouquerol’s criterion.^50^

Thermogravimetric and thermal differential (TG-DTG) analyses were conducted in an air stream with a NETZSCH STA 449F3 STA449F3A-1625-M analyzer (temperature ramp: 25 °C/10.0 (°C/min))/800 °C).

Infrared (FTIR) spectra were recorded in a PIS 100 spectrometer. The solid samples, mixed with KBr, were pressed into a pellet.

Transmission electron microscopy (TEM) measurements were carried out on a JEOL 200 keV instrument from the microscopy service of the Polytechnic University of Valencia. Energy dispersive X-ray (EDX) spectrometry analyses were performed at the same time to obtain the metallic composition of the MOF particles.

#### FTIR-CO Adsorption Study

IR spectra of adsorbed CO were recorded at low temperature (−165 °C) with a Nexus 8700 FTIR spectrometer using a DTGS detector and acquiring at 4 cm^–1^ resolution. An IR cell allowing *in situ* treatments in controlled atmospheres and temperatures from −165 to 500 °C has been connected to a vacuum system with a gas dosing facility. For IR studies, the samples were pressed into self-supported wafers and treated in vacuum (10^–5^ mbar) for 1.5 h at 250 °C. After activation, the samples were cooled down to −165 °C under dynamic vacuum conditions followed by CO dosing at 0.25 mbar. IR spectra were recorded after each dosage.

### Catalytic Tests

#### *N*-Alkylation of Aniline with Benzyl Alcohol

The *N*-alkylation reactions were performed in 2 mL of glass-vessel reactors equipped with a magnetic bar. Benzyl alcohol (0.60 mmol, 64.80 mg), aniline (0.60 mmol, 55.90 mg), and dodecane (0.22 mmol, 37.40 mg) as external standard and the corresponding solvent (1.35 mL) were added to each reactor containing the required amount of catalyst (12 mol % referred to the total metal content). The mixtures were placed in an aluminum heating block at 120 °C with magnetic stirring. Approximately 50 μL aliquots were taken at different times, diluted with ethyl acetate, and centrifuged. The supernatant obtained from batch reactions was analyzed using gas chromatography in an instrument equipped with a 25 m capillary column of 5% phenylmethylsilicone.

#### Kinetic Study for the *N*-Alkylation Reaction of Aniline with Benzyl Alcohol

The kinetic study reactions were performed in 2 mL glass-vessel reactors equipped with a magnetic bar. The corresponding amounts of benzyl alcohol (0.43, 0.33, 0.22, and 0.10 mol·L^–1^) and aniline (0.33, 0.30, 0.23, and 0.13 mol·L^–1^) in *o*-xylene (11.16 mmol, 1.35 mL), Hf-MOF-808_H_2_O (30.10 mg, 12 mol % Hf) as catalyst and dodecane (0.22 mmol, 37.40 mg) as external standard were added to each reactor. The mixtures were placed in an aluminum heating block at 120 °C with magnetic stirring. Approximately 50 μL aliquots were taken at different times, diluted with ethyl acetate, and centrifuged. The supernatant obtained from batch reactions was analyzed using gas chromatography in an instrument equipped with a 25 m capillary column of 5% phenylmethylsilicone.

#### Mechanistic Study for the *N*-Alkylation Reaction of Aniline with Benzyl Alcohol

The mechanistic experiments were performed in 2 mL glass-vessel reactors equipped with a magnetic bar. The corresponding amount of Hf-MOF-808_H_2_O (10.03 mg, 12 mol % Hf) was weighed in the reactors together with the different benzyl alcohols (PhCD_2_OH and PhCH_2_OD, 0.2 mmol), aniline (0.20 mmol, 18.6 mg) and/or *N*-benzylideneaniline (0.20 mmol, 36.2 mg), and *o*-xylene (0.45 mL). The mixtures were placed in an aluminum heating block at 120 °C under magnetic stirring during 5 h. The reaction mixtures were filtered with a PTFE syringe filter, diluted with toluene-*d*_8_ and analyzed by ^1^H NMR spectroscopy.

#### *N*-Alkylation Substrate Scope

The scope reactions were performed in 2 mL glass-vessel reactors equipped with a magnetic bar. The corresponding alcohols and amines (0.60 mmol of each of them), dodecane (0.22 mmol, 37.40 mg) as an external standard, and *o*-xylene (11.16 mmol, 1.35 mL) as solvent were added to each reactor containing the corresponding amount of Hf-MOF-808_H_2_O (30.10 mg, 12 mol % Hf). The mixtures were placed in an aluminum heating block at 120 or 140 °C with magnetic stirring. Approximately 50 μL aliquots were taken at different times, diluted with ethyl acetate, and centrifuged. The supernatant obtained from batch reactions was analyzed using gas chromatography in an instrument equipped with a 25 m capillary column of 5% phenylmethylsilicone.

#### Reuse of Hf-MOF-808_H_2_O in the *N*-Alkylation Reaction of Aniline with Benzyl Alcohol

The reuse reactions were performed in 2 mL glass-vessel reactors equipped with a magnetic bar. After the *N*-alkylation process was finished, the Hf-MOF-808_H_2_O was filtered off from the reaction crude and washed with *o*-xylene, ethyl acetate, methanol, and acetone. The material was then dried at 100 °C for 2 h in an oven before being reused in the next cycle. Between each of these reuse cycles, the catalyst was weighed to maintain constant catalytic charge/substrate and substrate/solvent ratios.

## Results and Discussion

### Synthesis and Characterization of MOF-Type Materials

Recently, the green preparation of different MOF-type materials, particularly UiO-66 and MOF-808, has been described via modulated hydrothermal synthesis (MHT).^49,51,52^ In these cases, water is used as the solvent during their crystallization, avoiding the use of *N*,*N*-dimethylformamide (DMF), a common and highly toxic solvent in the traditional solvothermal syntheses. Interestingly, it has been shown that the relative amount of defective −OH groups within the metallic nodes in MOFs can be substantially increased when the MOF preparation is carried out via MHT synthesis compared to the more classical solvothermal approach, a fact that unavoidably influences the adsorption and catalytic properties.^46^ Based on these recent results, we propose here to study different Zr- and Hf-MOF type materials, UiO-66 and MOF-808, prepared via both hydrothermal and solvothermal syntheses, as well as the catalytic implications that these different synthesis approaches would have for the tandem *N*-alkylation reaction between amines and benzyl alcohols.

**Figure 1:**
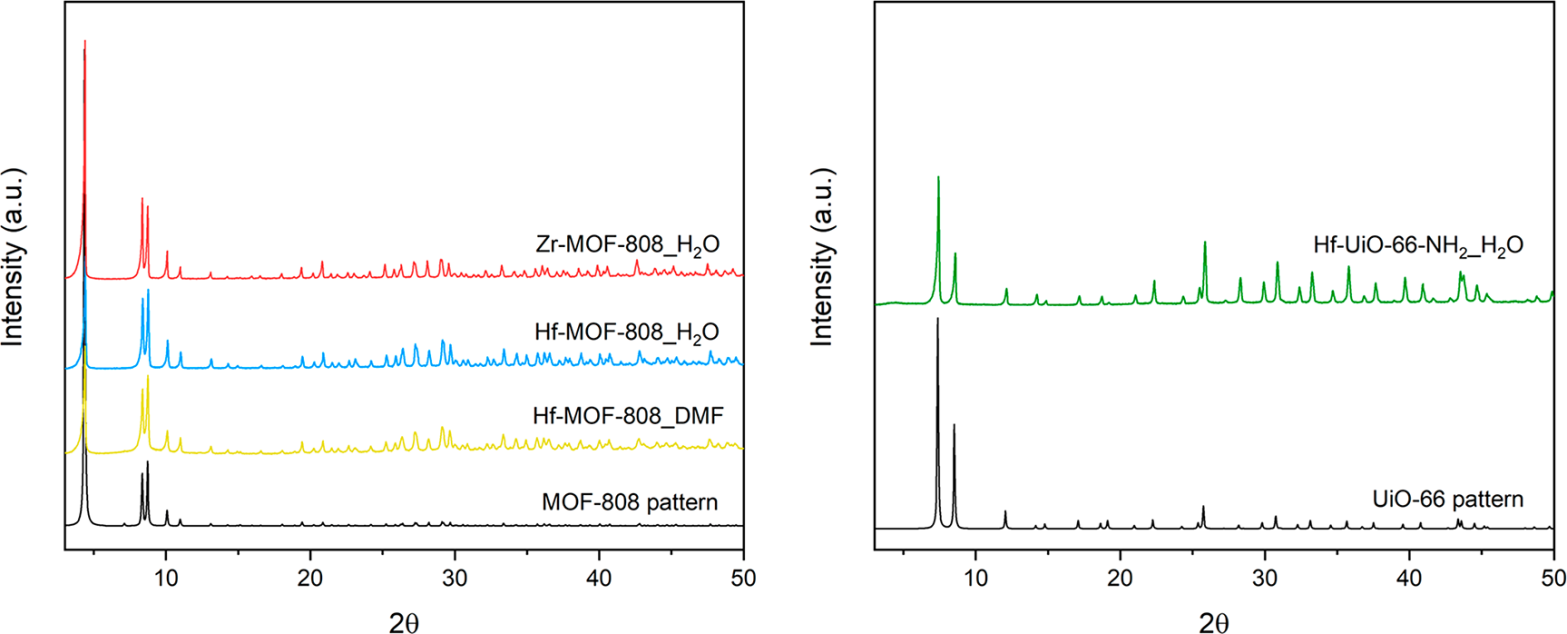
PXRD patterns of M-MOF-808 and Hf-UiO-66-NH_2_ synthesized via solvothermal (DMF) and hydrothermal (H_2_O) methods.

The detailed synthesis descriptions of the different metal-containing MOFs can be found in the Experimental Section. Those materials have been adequately characterized by different techniques to unravel their physicochemical properties. The powder X-ray diffractograms of the different solids reveal the formation of the pure crystalline MOF-808 or UiO-66 phases in all cases (see Figure 1).

**Figure 2:**
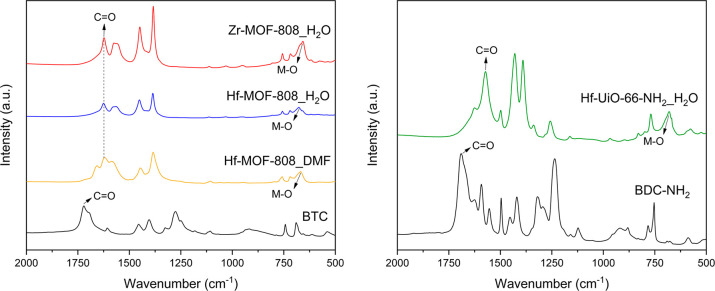
FTIR spectra of M-MOF-808 and Hf-UiO-66-NH_2_ synthesized via solvothermal (DMF) and hydrothermal (H_2_O) methods.

The interaction of the organic ligands with the different metallic clusters has been studied by FTIR spectroscopy (see Figure 2). The spectra of the different MOFs present a clear shift of the ∼1700 cm^–1^ signal assigned to the carbonyl stretch C=O of the free carboxylic acid group toward lower frequencies, as well as the appearance of a signal centered at ∼700–600 cm^–1^, which is assigned to the vibration mode frequency of the M–O linkages.

**Figure 3:**
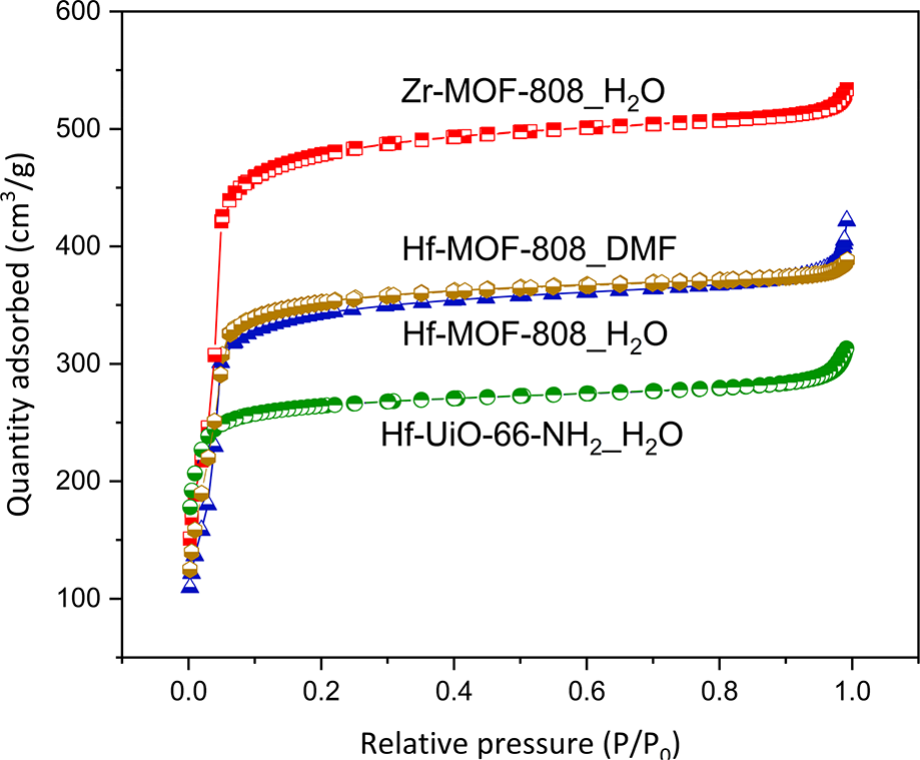
N_2_ adsorption–desorption isotherms of M-MOF-808 and Hf-UiO-66-NH_2__H_2_O synthesized via solvothermal (DMF) and hydrothermal (H_2_O) methods.

The textural properties of the as-obtained MOF samples have been analyzed from the N_2_ adsorption–desorption isotherms (see Figure 3). The entire MOFs exhibited a type-I isotherm with a minor hysteresis between adsorption and desorption branches.^53^

The surface area calculated by the BET method, following Rouquerol’s criterion, for the Zr-MOF-808_H_2_O sample is considerably higher than for the Hf counterpart (see Table 1, entries 1 and 2, respectively), a fact that can be explained by the higher density of the Hf-MOF-808_H_2_O sample.^54^ Moreover, the Hf-MOF-808 prepared via hydrothermal and solvothermal syntheses presents similar BET surface areas (see Table 1, entries 2 and 3, respectively). Finally, the Hf-UiO-66-NH_2__H_2_O shows a measured BET surface area analogous to those reported previously for this material in the literature,^49^ but this value is lower compared to Hf-MOF-808 samples (see Table 1, entry 4). The higher cluster connectivity of UiO-66 (12-connected) together with the smaller pore size (8 and 11 Å) of this structure compared to MOF-808, which presents a 6-connectivity and adamantane-type apertures of ∼18.4 Å, would explain this difference.

**Table 1:** Physicochemical Properties of the Different Zr- and Hf-MOFs

entry	sample	C^a^ (%)	H^a^ (%)	N^a^ (%)	M^b^ (%)	BET surf. area (m^2^/g)	micro. area (m^2^/g)	microp. vol. (cm^3^/g)
1	Zr-MOF-808_H_2_O	22.8	3.0	-	26.5	1540	1475	0.7
2	Hf-MOF-808_H_2_O	15.4	3.9	-	44.5	1109	1052	0.5
3	Hf-MOF-808_DMF	17.9	2.1	2.1	36.2	1135	1093	0.5
4	Hf-UiO-66-NH_2__H_2_O	22.2	3.6	2.4	37.2	848	812	0.4

a Determined by elemental analysis.

b Determined by ICP-OES analysis.

The chemical composition and stability of the hydrothermal and solvothermal catalysts have been studied by ICP, elemental analysis, and TGA analysis (Table 1 and Figure S1). The M/C molar ratios obtained for the Hf and Zr-MOFs are consistent with their molecular formulas. Moreover, the TGA analysis reveals that all MOFs have high thermal stability under oxidant atmospheres and would be stable up to 350 °C (see Figure S1).

**Figure 4:**
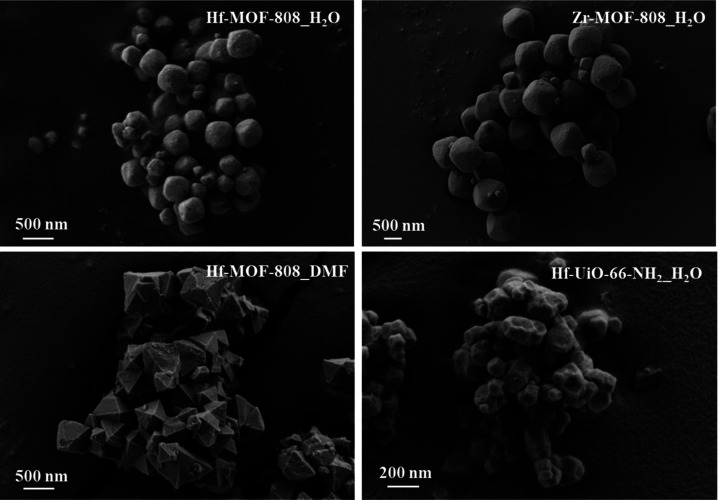
FE-SEM images of M-MOF-808 and Hf-UiO-66-NH_2_ synthesized via solvothermal (DMF) and hydrothermal (H_2_O) methods.

Finally, the morphology of the MOF materials has been evaluated by field-emission scanning electron microscopy (FE-SEM, see Figure 4). The samples prepared by the MHT synthesis present a quasi-spheroidal morphology (see M-MOF-808_H_2_O and Hf-UiO-66-NH_2__H_2_O in Figure 4), while the Hf-MOF-808 prepared by the solvothermal approach shows an octahedral morphology (see Hf-MOF-808_DMF in Figure 4). The different crystal morphologies achieved clearly indicate the influence of the applied synthetic method on the final nucleation and crystallization processes.^55^ In addition, Hf-MOF-808_H_2_O material shows a smaller particle size (∼300 nm, see Figure 4) compared to Zr-MOF-808_H_2_O (∼900 nm, see Figure 4). Despite Hf and Zr exhibiting similar ionic radii, it has been broadly described in coordination chemistry that Hf performs in a sluggish way compared to Zr,^56^ maybe influencing the overall crystal growth of the MOF particles.

### *N*-Alkylation Reaction of Aniline with Benzyl Alcohol

**Scheme 2:**
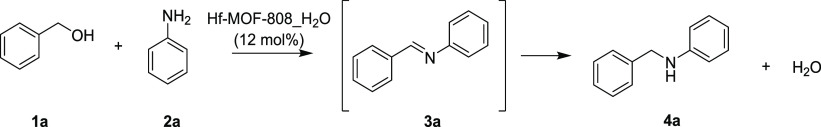
*N*-Alkylation Reaction of Aniline **2a** with Benzyl Alcohol **1a** to Afford the *N*-Benzylaniline Product **4a**

The different Zr- and Hf-containing MOFs have been tested as catalysts for the *one-pot* synthesis of the *N*-benzylaniline (**4a**) with aniline (**2a**) and benzyl alcohol (**1a**) as starting materials (see Scheme 2). A preliminary optimization of the reaction conditions has been first carried out, in which the metal loading of Hf-MOF-808_H_2_O and the reaction temperature were varied. The highest yield toward the desired *N*-benzylaniline has been obtained at 120 °C and with 12 mol % Hf after 2 h of reaction (∼85%, see blue triangles in Figure S2), where complete benzyl alcohol conversion has been reached. Moreover, the catalyst loading could be reduced to even 8 mol % to achieve similar **4a** yield after 5 h (see black squares in Figure S2A). However, neither at 110 °C nor at 100 °C does the benzyl alcohol conversion exceed 20% after 2 h of reaction (see black squares and red circles in Figure S2B, respectively). Furthermore, the kinetic curves show a sigmoidal shape that indicates the presence of an induction period where the active phase of the catalyst is being formed (see Figure S2). This initial induction period could be minimized if Hf-MOF-808_H_2_O is previously activated with benzyl alcohol for 2 h at 120 °C, and after that period, an equimolar amount of aniline is then injected (see Figure S3). A complete benzyl alcohol conversion and high *N*-benzylaniline yield (∼86%) are achieved after only 0.75 h at 120 °C (see Figure S3), suggesting that benzyl alcohol would have an important role in the *N*-alkylation reaction mechanism.

An additional optimization of the *N*-alkylation reaction conditions using Hf-MOF-808_H_2_O as catalyst has been performed by studying different solvents at 120 °C. From this second set of experiments, polar solvents, as DMF and DMSO, do not afford the corresponding **4a** product after 2 h (see Table S1, entries 1 and 2, respectively). The same trend is observed when moderate polar solvents, such as butyl acetate or 2-methoxyethanol, which contain carbonyl and alcohol groups in their structures, respectively, were used (see Table S1, entries 3 and 4, respectively). The undesired adsorption of these solvent molecules on the metallic nodes of the MOF catalyst could block the access of the reaction substrates to the active sites. The best catalytic results have been obtained using 1,2-dichlorobenzene and *o*-xylene as solvents (see Table S1, entries 5 and 6, respectively). Particularly, *o*-xylene shows the highest catalytic activity after 2 h at 120 °C with an ∼85% yield of the *N*-benzylaniline product (see Table S1, entry 6). The different activity shown as a function of the solvent polarity and structure could be tentatively attributed to the chemical environment created around the active sites. This behavior has been observed previously in enzymes^57^ and other inorganic solids, such as zeolites,^58^ where well-defined confined spaces promote high activities and selectivities toward the desired product. In our case, the nonpolar solvent could facilitate the promotion of hydrophobic or cage effects since the reagent substrates have polar groups within their structures. The nonpolar environment would allow the pre-organization of the reactants close to the active sites, which would be electronically decompensated due to the defects in the metal–organic framework, enabling the interaction between them and, consequently, the formation of the desired *N*-alkylation reaction product.

At this point, the *one-pot* reaction between aniline **2a** and benzyl alcohol **1a** has been studied under the above optimized reaction conditions (*T* = 120 °C and *o*-xylene as solvent), using the different Zr- and Hf-metal–organic materials synthesized and characterized in this work, together with other homogeneous and heterogeneous catalysts (see Table 2).

**Table 2:** *N*-Alkylation Reaction between Benzyl Alcohol and Aniline Employing Different Catalysts^a^

entry	sample	alcohol conversion (%)^b^	**3a** (%)^b^	**4a** (%)^b^
1	Hf-MOF-808_H_2_O	98.6	0.6	85.3
2	Hf-MOF-808_DMF	56.5	17.5	17.2
3	Zr-MOF-808_H_2_O	99.0	1.5	83.2
4	Hf-UiO-66-NH_2__H_2_O	22.6	1.5	0.9
5	HfO_2_	6.4	0.0	0.4
6	HfCl_4_	24.1	0.3	0.4
7	HfOCl_2_·8H_2_O	8.6	0.0	0.3
8	ZrO_2_-np	4.5	0.0	0.8
9	H_3_BTC	4.4	0.0	0.4

a Reaction conditions: benzyl alcohol **1a** (0.60 mmol), aniline **2a** (0.60 mmol), catalyst (12 mol % M), *o*-xylene (11.16 mmol, 1.35 mL), dodecane as external standard (0.22 mmol, 37.40 mg), *T* = 120 °C, 2 h.

b Conversion and yield were determined by gas chromatography.

The two M-MOF-808_H_2_O (M: Hf and Zr) materials, which have been prepared under hydrothermal conditions, present the highest catalytic performance for the *N*-alkylation reaction, with complete alcohol conversion after 2 h (see Table 2, entries 1 and 3, respectively). Selectivities of ∼85% toward *N*-benzylaniline **4a** have been determined for both materials, detecting only traces of the corresponding imine **3a**.

**Figure 5:**
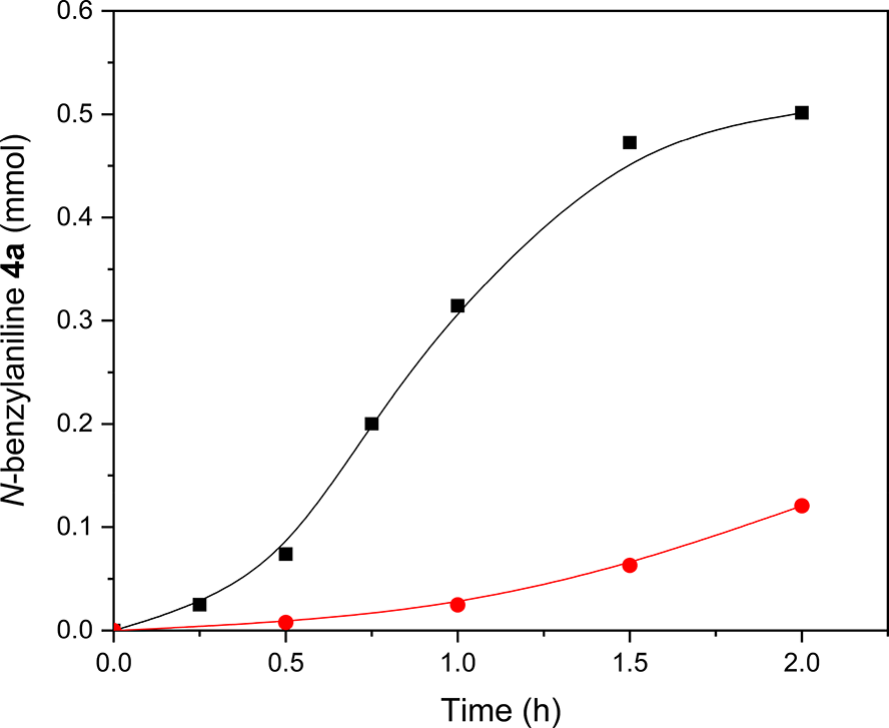
*N*-alkylation reaction between benzyl alcohol and aniline catalyzed by Hf-MOF-808_H_2_O (■) and Hf-MOF-808_DMF (●).

However, the Hf-MOF-808_DMF catalyst prepared via solvothermal synthesis shows much lower catalytic activity for the *N*-alkylation reaction compared to the two M-MOF-808 prepared via hydrothermal synthesis (see entry 2 in Table 2 and Figure 5).

**Figure 6:**
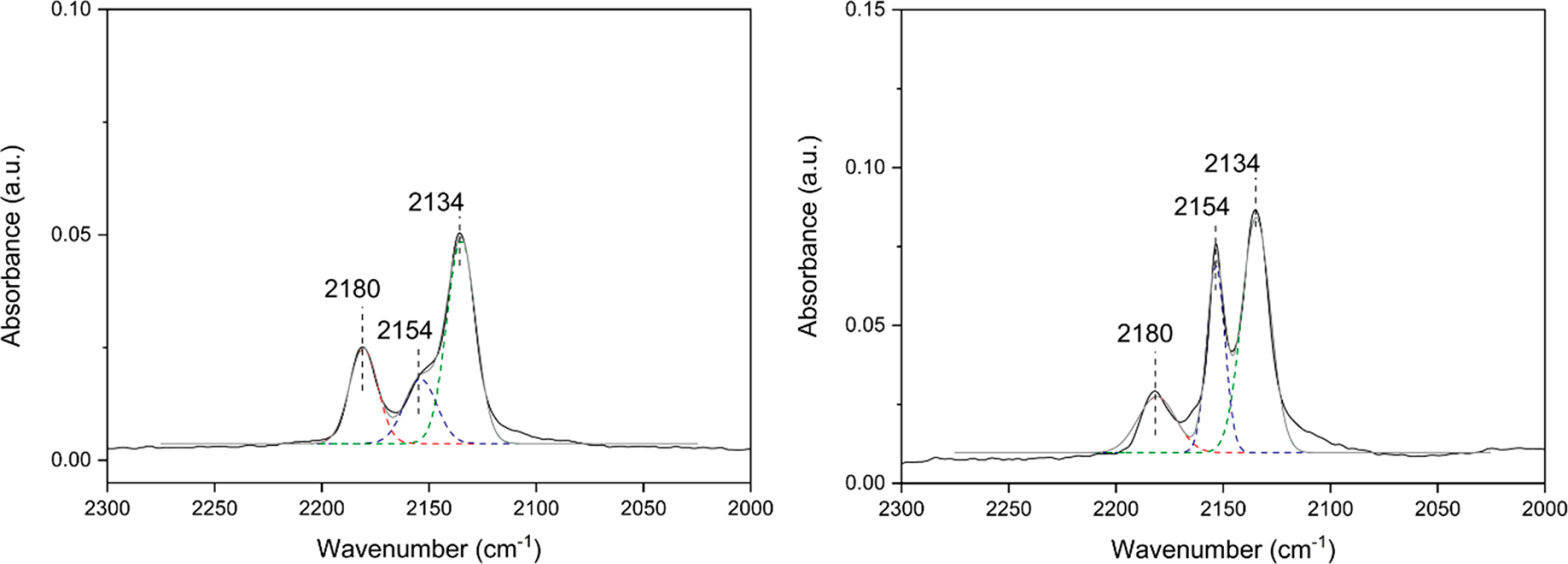
FT-IR spectra of CO adsorption at −165 °C at 0.25 mbar on Hf-MOF-808_DMF (left panel) and Hf-MOF-808_H_2_O (right panel).

Recently, we have determined that the Hf-MOF-808 synthesized via the hydrothermal method contains a much higher concentration of defective −OH sites than the same sample prepared via the solvothermal approach, which undoubtedly plays an important role in the final activity of this material for specific catalytic reactions.^42,46^ Thus, the different catalytic behaviors observed between the MOF-808 samples prepared via modulated hydrothermal and solvothermal approaches could be tentatively assigned to the fact that the defective −OH species would facilitate the hydride formation due to the proton uptake of the alcohol group (step 1 in Scheme 1). To determine the relative concentration of −OH sites, FTIR spectroscopic studies of CO as the probe molecule have been carried out. For both Hf-MOF-808 samples, IR peaks at 2134, 2154, and 2180 cm^–1^ assigned to CO physisorbed and interacting with slight acid −OH groups and Hf Lewis acid sites, respectively, were detected (see Figure 6).^39^

However, the relative ratio between the integrated signals referred to Brønsted and Lewis acid sites (2154 and 2180 cm^–1^, respectively) is at least two times higher for the Hf-MOF-808_H_2_O sample than for the Hf-MOF-808_DMF, suggesting that the relative concentration of defective −OH sites is considerably higher in the Hf-MOF-808 synthesized via the hydrothermal method (see Figure 6).

On the other hand, another MOF-type structure, UiO-66, containing Hf, has been prepared under hydrothermal syntheses. Hf-UiO-66-NH_2__H_2_O, which has an additional basic group that may be able to capture/stabilize the proton of the benzyl alcohol molecule and, then, favor its dehydrogenation, does not show any detectable catalytic activity for the *N*-alkylation reaction (see Table 2, entry 4). These differences observed between both types of MOFs, MOF-808 and UiO-66, may be ascribed to higher diffusional limitations along the UiO-66 structure, since this framework not only has higher theoretical organic ligand connections per metallic node than MOF-808 (12 and 6, respectively),^59^ but also smaller pore sizes (8–11 and 18.4 Å for UiO-66 and MOF-808, respectively).

Neither the heterogeneous catalyst bulk HfO_2_ nor the homogeneous HfCl_4_ and HfOCl_2_·8H_2_O show any activity for the *N*-alkylation reaction (see Table 2, entries 5, 6, and 7, respectively). In addition, ZrO_2_-np (<100 nm) nanoparticles, even when they present remarkably smaller particle sizes than HfO_2_ and, thus, larger external surface areas to facilitate the interaction with the reagents do not present an appreciable catalytic activity (see Table 2, entry 8). In the same way, H_3_BTC has been studied as a homogeneous catalyst, presenting low catalytic activity (see Table 2, entry 9) and, then, ruling out the possible contribution of the organic ligands during the catalytic process.

### Kinetic and Mechanistic Studies for the *N*-Alkylation Reaction of Aniline with Benzyl Alcohol

To better understand the mechanism of the *N*-alkylation reaction when Hf-MOF-808_H_2_O is employed as catalyst, different kinetic and mechanistic studies have been carried out. The three reaction steps required in the *N*-alkylation reaction are described in Scheme 1, where it was assumed that each one could, in principle, be the overall rate-determining step with the other two reactions being in equilibrium. Following this, the resultant kinetic rate equations are presented in Table S2.^10^

**Figure 7:**
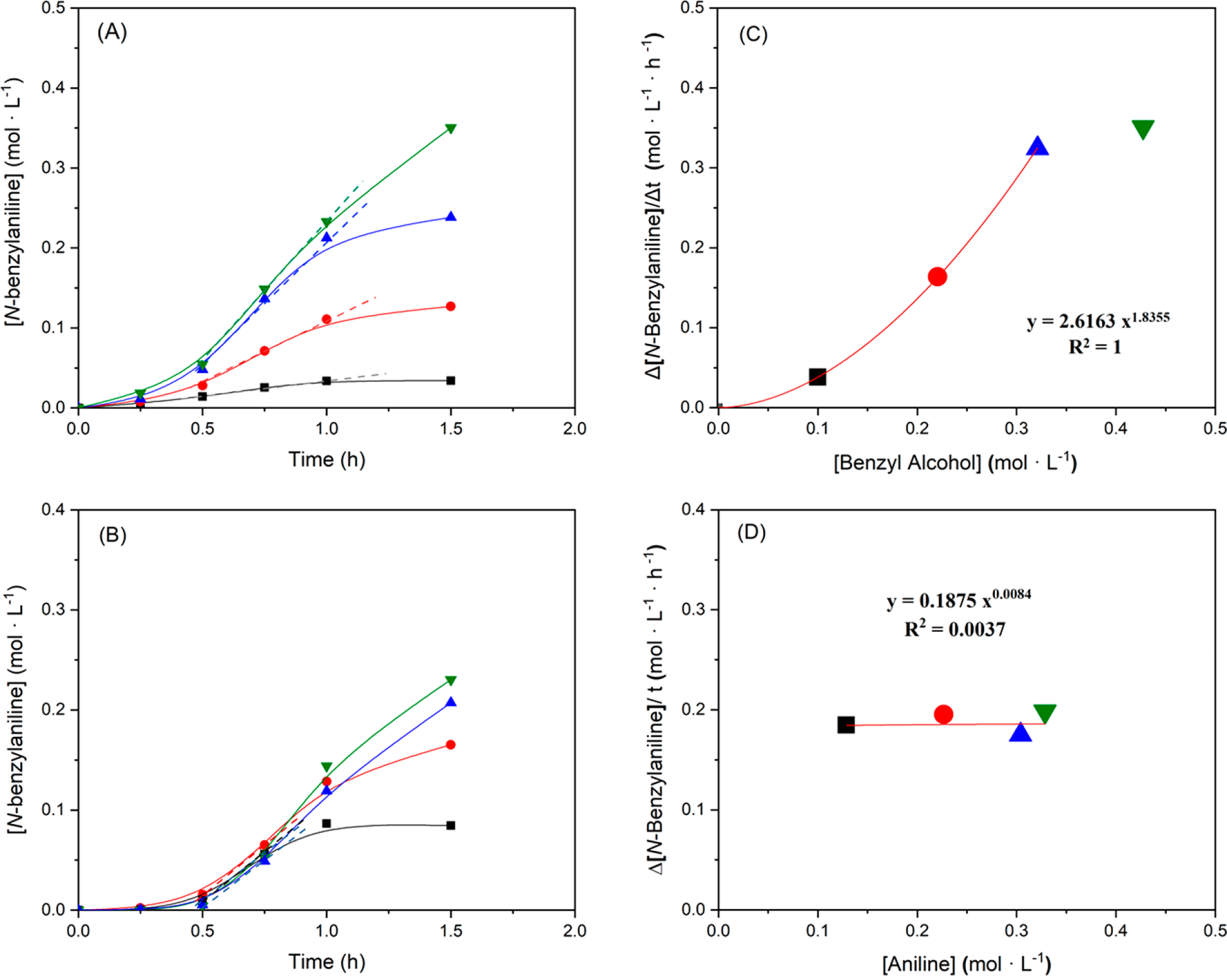
Kinetic study for the *N*-alkylation reaction of aniline **2a** with benzyl alcohol **1a** using Hf-MOF-808_H_2_O as catalyst (12 mol % M), *o*-xylene (11.16 mmol, 1.35 mL), dodecane as an external standard (0.22 mmol, 37.40 mg), *T* = 120 °C, 1.5 h. Conversion and yield were determined by gas chromatography. (A) Variation of the concentration of benzyl alcohol **1a** (0.43 (▼), 0.33 (▲), 0.22 (●), and 0.10 mol·L^–1^ (■)). (B) Variation of the concentration of aniline **2a** (0.33 (▼), 0.30 (▲), 0.23 (●), and 0.13 mol·L^–1^ (■)). (C) Reaction order of benzyl alcohol **1a**. (D) Reaction order of aniline **2a**.

To discriminate among the three kinetic expressions, the initial concentration of benzyl alcohol was varied (0.43, 0.33, 0.22, and 0.10 mol·L^–1^) while the concentration of aniline was kept constant (0.43 mol·L^–1^) (see Figure 7A), and the initial reaction rates were measured. The same procedure was applied but varying the concentration of aniline (0.33, 0.30, 0.23, and 0.13 mol·L^–1^) and maintaining constant the concentration of benzyl alcohol (0.43 mol·L^–1^) (see Figure 7B).

The entire kinetic experiments show sigmoidal curves (see Figure 7A,B), clearly indicating the presence of an induction period in all cases. To evaluate the dependence of the initial reaction rate with reactants, the initial reaction rates have been estimated, without considering the induction period, (see slopes in Figure 7A,B). This kinetic analysis suggests a dependence of the initial reaction rate with the concentration of benzyl alcohol (see Figure 7C), where the initial reaction rate increases exponentially with the concentration of benzyl alcohol until reaching a maximum concentration. This would explain why the induction period of the kinetic curves could be suppressed with a previous thermal treatment of the Hf-MOF-808_H_2_O catalyst with benzyl alcohol (120 °C for 2 h, see Figure S3), facilitating the *in situ* benzaldehyde formation before introducing aniline. In contrast, the reaction order of aniline is ∼0 (see Figure 7D). Thus, according to the rate equations shown in Table S2, only the benzyl alcohol dehydrogenation reaction (see eq 1) depends exclusively on the concentration of benzyl alcohol, indicating that this could be the rate-limiting step. Similar theoretical and experimental results supporting the fact that the benzyl alcohol dehydrogenation reaction is the rate-limiting step for the *N*-alkylation reaction have been recently reported using metal-containing zeolites as catalysts.^30^

**Figure 8:**
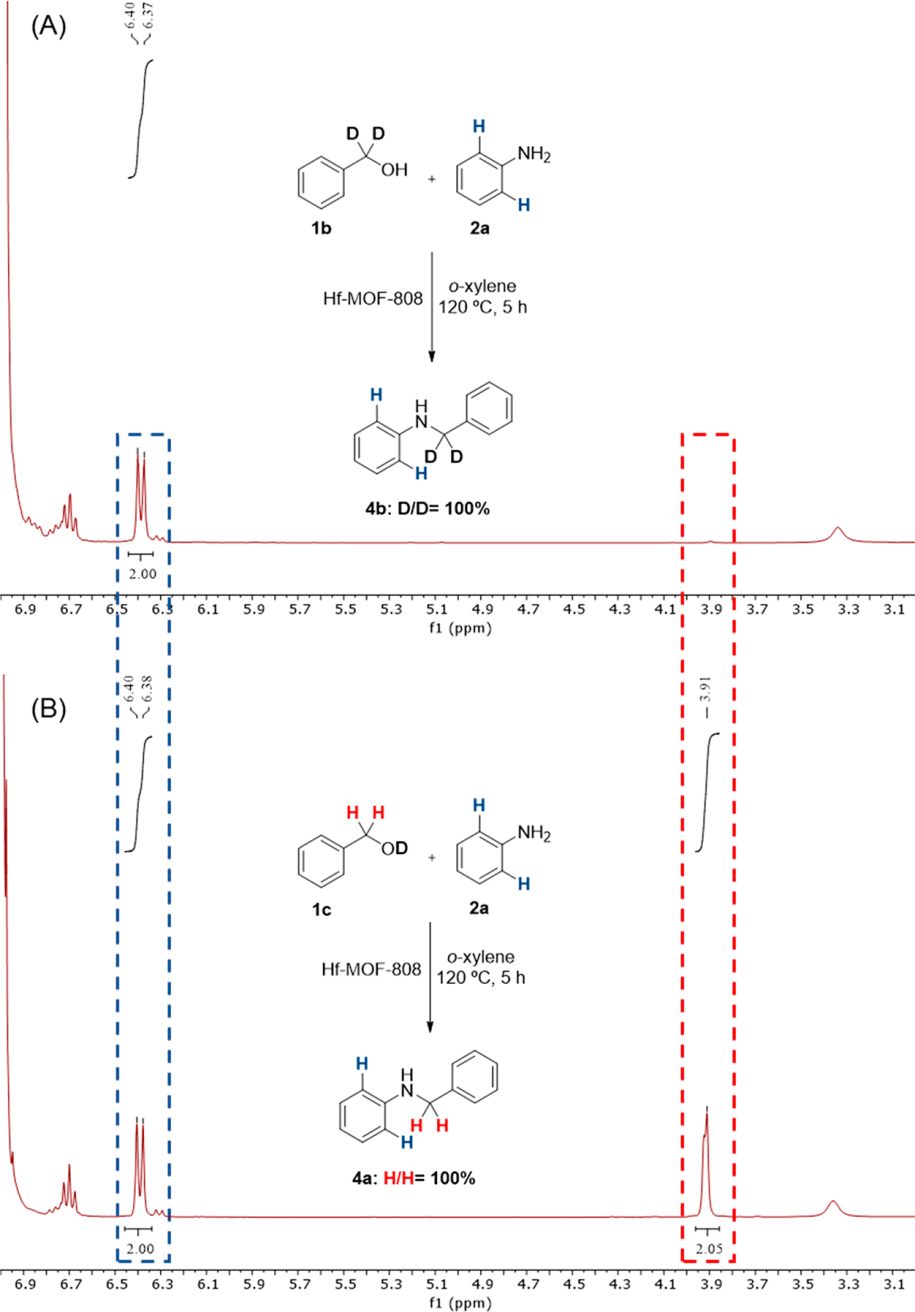
Mechanistic reactions for *N*-alkylation of aniline **2a** with isotopically labeled benzyl alcohols (A) PhCD_2_OH (**1b**) and (B) PhCH_2_OD (**1c**).

Subsequently, we conducted deuterium-labeling experiments using ^1^H NMR spectroscopy (see Figure 8). To unravel the nature of the metal hydride, two isotopically labeled benzyl alcohols **1b** and **1c** were employed as starting materials in the *N*-alkylation reaction (see Figure 8A,B, respectively). The obtained ^1^H NMR spectra confirm that the hydrogen borrowed by the catalyst is the one contained in the methylene position of benzyl alcohol and does not proceed from the hydroxyl group of benzyl alcohol.

**Figure 9:**
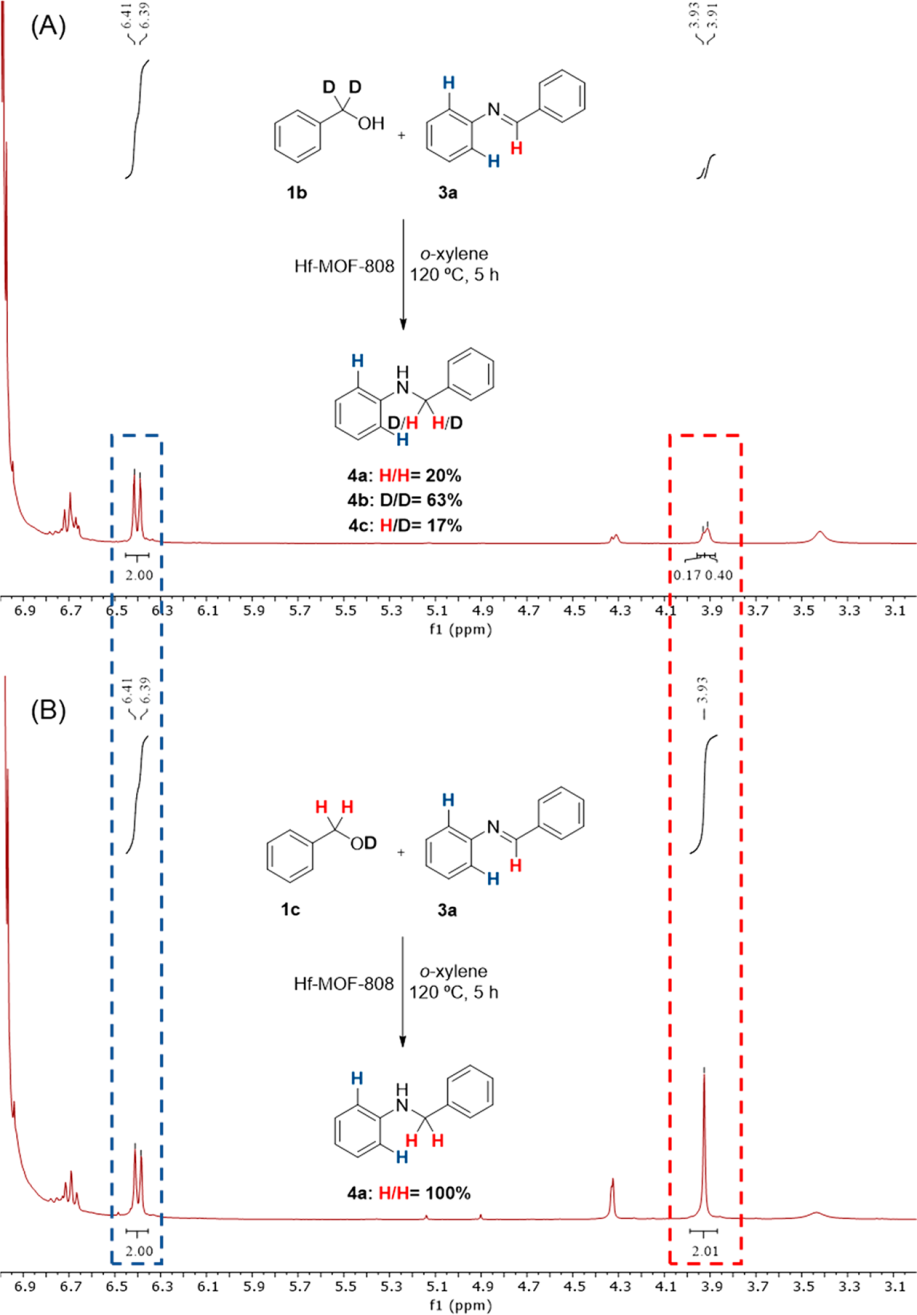
Mechanistic reactions for the hydrogenation of *N*-benzylideneaniline **3a** with isotopically labeled benzyl alcohols (A) PhCD_2_OH (**1b**) and (B) PhCH_2_OD (**1c**).

To corroborate this hypothesis, the hydrogenation of *N*-benzylideneaniline **3a** with the two isotopically labeled benzyl alcohols **1b** and **1c** has been studied (see Figure 9A,B, respectively). It can be observed in the ^1^H NMR spectra that the hydrogenation of the imine **3a** with PhCD_2_OH (**1b**) also favors the fully incorporation of deuterium in the methylene position of the amine product **4b** (see Figure 9A), whereas the deuterium from de hydroxyl group of **1c** is not able to hydrogenate the imine (see Figure 9B).

This evidence suggests that the Hf Lewis acid sites are able to borrow the hydrogen from the methylene position of benzyl alcohol to hydrogenate the imine group. Finally, the influence of the isotopic exchange in the methylene position of benzyl alcohol on the initial reaction rate has been studied, confirming an isotopic effect of *k*_H_/*k*_D_ = 3.03 (see Figure S4). This isotopic effect result, complemented with the previous kinetic experiments, would further suggest that the alcohol dehydrogenation must be involved in the rate-determining step of the catalytic cycle.

**Scheme 3:**
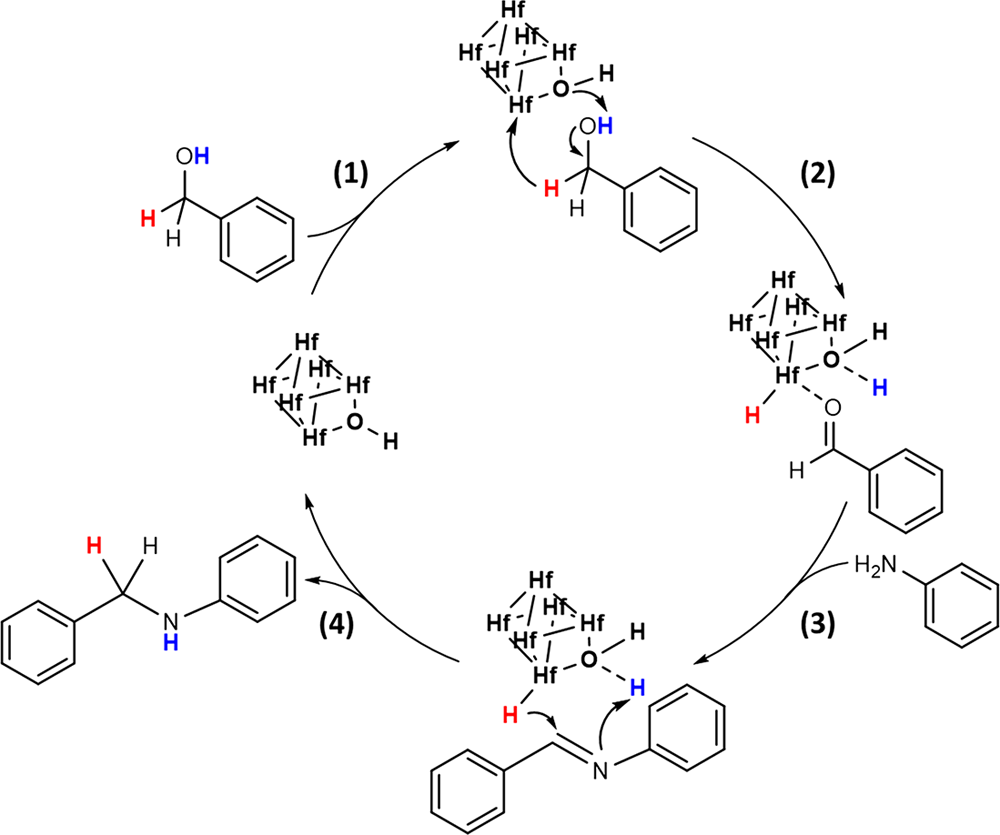
Hydrogen *Auto*-transfer Mechanism for the *N*-Alkylation Reaction of Aniline with Benzyl Alcohol to Afford the *N*-Benzylaniline Product with Hf-MOF-808_H_2_O as Catalyst

With all the information obtained from kinetic and mechanistic studies, we tentatively propose a possible catalytic route for the *N*-alkylation of aniline with benzyl alcohol using Hf-MOF-808_H_2_O as catalyst based on the borrowing hydrogen pathway (BH) (see Scheme 3):^5^ (1) The first step consists in the alcohol deprotonation by the metallic cluster with the defective −OH group and the consequent dehydrogenation. (2) After the alcohol deprotonation and dehydrogenation processes, a benzaldehyde molecule is produced at the same time that the Hf-hydride is generated. (3) Subsequently, the benzaldehyde adsorbed on the Hf site, which increases its electrophilicity and, therefore, its reactivity, instantly undergoes a nucleophilic addition by the amino group of the aniline, giving rise to the corresponding imine. This process involves the loss of a water molecule. (4) Finally, the Hf-hydride generated in the first stage hydrogenates the imine group at the same time that the proton captured by the defective −OH group is taken by the imine nitrogen to give rise to the corresponding *N*-benzylaniline product, while the active site is regenerated.

### *N*-Alkylation Substrate Scope

To determine the generality of the method developed for the *N*-alkylation reaction with Hf-MOF-808_H_2_O as catalyst, different benzyl alcohols and substituted anilines have been studied as starting materials (see Table 3).

**Table 3:**
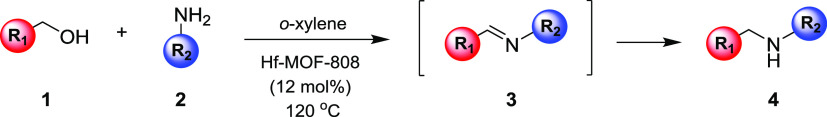

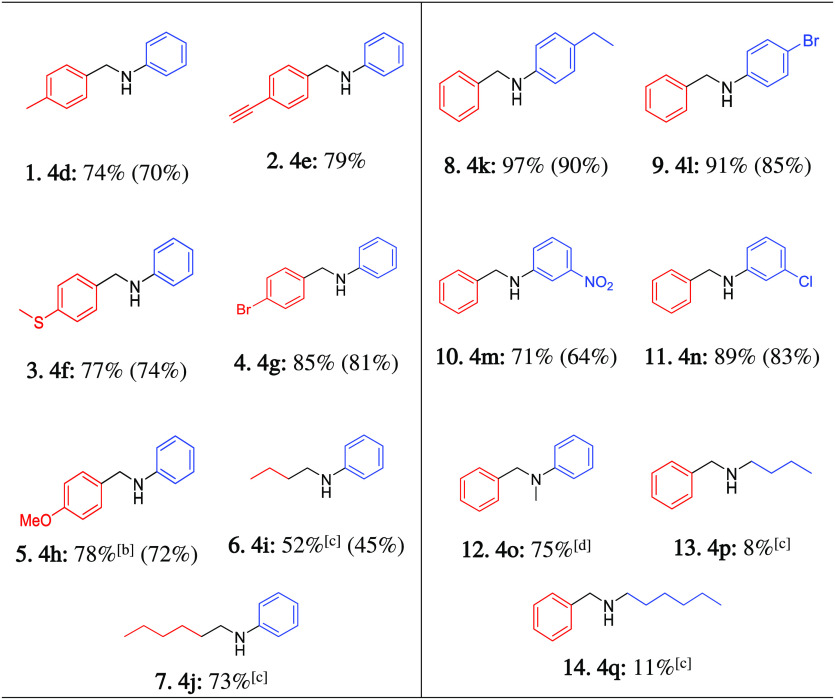
*N*-Alkylation Substrate Scope Using Different Amines and/or Alcohols^a^

a Reaction conditions: alcohol (0.60 mmol), amine (0.60 mmol), Hf-MOF-808_H_2_O (12 mol % Hf), *o*-xylene (11.16 mmol, 1.35 mL), dodecane as external standard (0.22 mmol, 37.40 mg), *T* = 120 °C, 3 h. Yield determined by gas chromatography. Isolated yield in parentheses.

b 1.5 h.

c *T* = 140 °C, *P*_air_ = 5 bar, 23 h.

d 23 h.

Under the optimal conditions, the method appears to be applicable in terms of catalytic performance and selectivity toward the desired *N*-benzylaniline products when the nature and position of the substituents on the benzyl alcohol (see Table 3, entries 1, 2, 3, 4, and 5) and aniline ring (see Table 3, entries 8, 9, 10, and 11) are varied. Interestingly, halogen substituents, including Br and Cl (see Table 3, entries 4, 9, and 11), are well tolerated, and dehalogenation processes have not been detected. In the same way, this catalytic system is compatible with starting materials presenting reducible groups, such as triple or nitro bonds (see Table 3, entries 2 and 10, respectively), where the hydrogenation of these groups has not been observed. Even the challenging sulfur-containing benzyl alcohol can lead to the desired product in good yields (see Table 3, entry 3).

It is worth noting that when 4-methoxybenzyl alcohol is employed for the *N*-alkylation reaction with aniline (see Table 3, entry 5), the complete conversion value is achieved after 1.5 h. On the other hand, the product yield drops to 71% when a strongly deactivating group, as the nitro group, is introduced in the *meta* position of the aniline ring (see Table 3, entry 10). This could be because the amino group becomes less nucleophilic when a substituent with an electron withdrawing group is present. The use of a secondary amine, also less active than a primary amine, requires longer reaction times to achieve complete conversion (see Table 3, entry 12). Moderate yields have been obtained when aliphatic alcohols such as *n*-butanol and *n*-hexanol have been employed as starting sources (see Table 3, entries 6 and 7, respectively), a fact that can be assigned to the formation of an unconjugated aldehyde, and, therefore, the corresponding imine with less conjugation than *N*-benzylideneaniline should be less stable. However, this methodology is not efficient when working with aliphatic amines (see Table 3, entries 13 and 14). Considering all these results, we can affirm that the method developed in the present work is effective for its use for a large variety of benzyl alcohols and substituted anilines, and in conjunction with our previous work,^27^ would allow covering a very large range of substrates, including aliphatic and aromatic reagents.

### Reuse of Hf-MOF-808_H_2_O in the *N*-Alkylation Reaction of Aniline with Benzyl Alcohol

One of the fundamental aspects of heterogeneous catalysts that differentiates them from their homogeneous counterparts is their easy extraction from the reaction medium and the possibility of being reused in successive catalytic cycles. Thus, the heterogeneity and stability of Hf-MOF-808_H_2_O under the optimized reaction conditions for the *N*-alkylation of aniline **2a** with benzyl alcohol **1a** have been studied.

**Figure 10:**
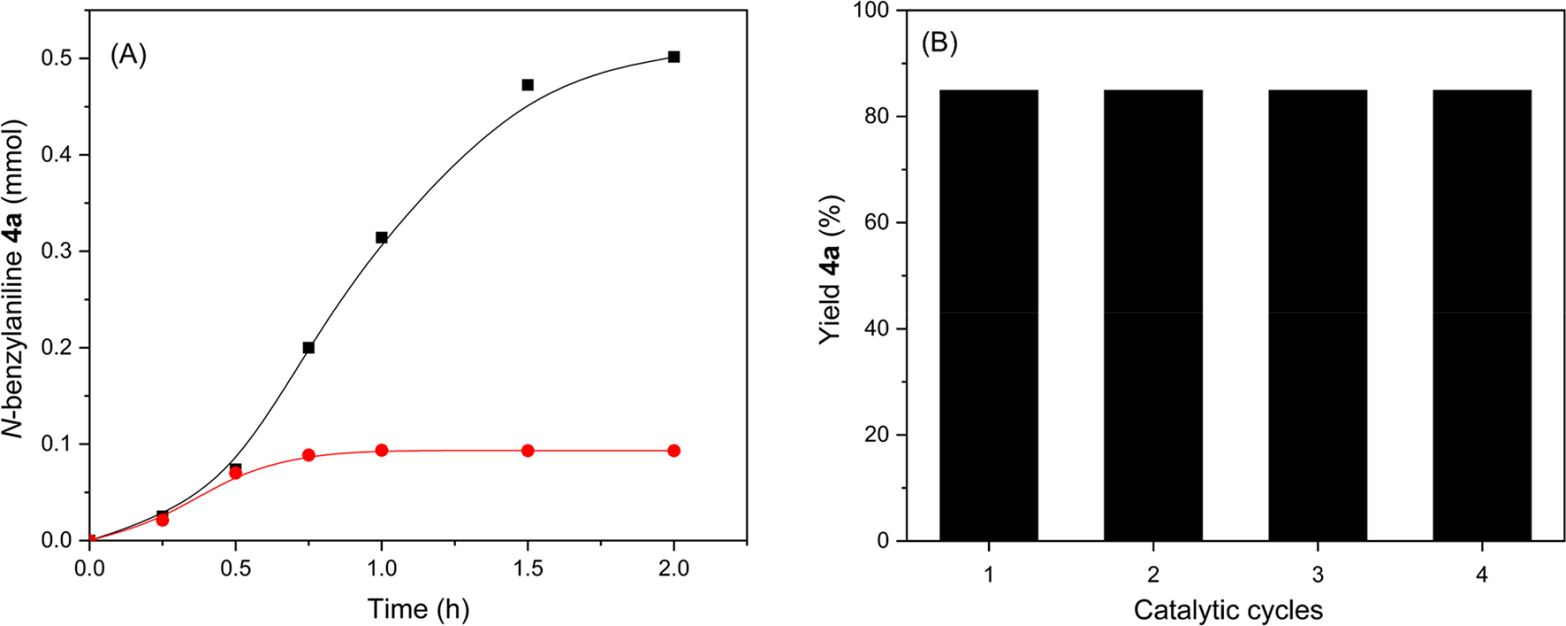
(A) Hot filtration test and (B) recyclability test for Hf-MOF-808_H_2_O catalyst after 4 successive cycles for the *N*-alkylation reaction of aniline with benzyl alcohol, where a 2 h reaction is performed for each catalytic cycle.

To do this, the hot filtering technique has been first carried out after 30 min (see Figure 10A). After that, the evolution of the reaction has been followed by gas chromatography until 2 h. During this period, no increment in the yield of product **4a** has been observed. These results reflect the catalyst heterogeneity and, therefore, the absence of leaching processes of active species from the catalyst to the reaction media. Furthermore, Hf-MOF-808_H_2_O could be reused successfully up to 4 consecutives catalytic cycles without a significant decrease of the *N*-benzylaniline **4a** yield (see Figure 10B).

The recovered material after the successive catalytic cycles has been analyzed by several techniques including powder X-ray diffraction, FE-SEM and TEM/EDX microscopy, solid-state ^13^C MAS NMR and FTIR spectroscopies, TGA, and elemental analysis. The PXRD pattern reveals the integrity of the crystalline structure of MOF-808, while FE-SEM images show the maintenance of the crystal morphology (see Figure S5). Moreover, through TEM/EDX analysis, similar Hf contents and distribution have been obtained for the fresh and recovered material (25 and 23%, respectively), also corroborating the structural integrity of the MOF sample under the studied reaction conditions (see Figure S6).

However, an increment of the organic content is observed by TGA and elemental analysis in the recovered Hf-MOF-808 material (see Figure S7 and Table S3, respectively), suggesting that some organic substrates are retained within the MOF-type catalyst. To elucidate the nature of the adsorbed products, solid-state ^13^C MAS NMR and FTIR spectroscopies have been employed. The characteristic bands of the *N*-benzylaniline product are clearly detected in both cases (see Figures S8 and S9, respectively). Moreover, ^1^H NMR and GC-MS spectra of the digested Hf-MOF-808_H_2_O sample with a mixture of D_2_SO_4_/DMSO-*d*_6_ elucidate the *N*-benzylaniline nature of the adsorbed organic substrate (see Figures S10 and S11, respectively).

Finally, through the elemental analysis of the reused solid, an additional 1.16% of N was detected (Table S3). Taking into account that the extra-nitrogen amount must be associated with the *N*-benzylaniline product adsorbed during the catalytic process, the overall real product yield value obtained toward **4a** would be 92% instead of 85%.

## Conclusions

The results presented in this work reveal the excellent ability of the hydrothermally synthesized Hf-MOF-808, denoted as Hf-MOF-808_H_2_O, to catalyze the *N*-alkylation reaction of aniline with benzyl alcohol. The presence of defective −OH groups on the metallic nodes together with the Hf Lewis acid sites enhances the catalytic behavior of this material for the *N*-alkylation reaction. Through kinetic and deuterium-labeling experimental studies, it has been demonstrated that the mechanism occurs via a borrowing hydrogen pathway, in which the dehydrogenation step has been determined as the rate-determining step. Moreover, the method described in this study is adequate for a wide range of aniline and benzyl alcohol derivates. Finally, Hf-MOF-808_H_2_O could be used in at least four consecutive catalytic cycles without observing a significant catalytic activity loss.

## Supporting Information Available

TGA analysis of the catalyst samples used; optimization of the metal loading, reaction temperature, and the solvent employed in the *N*-alkylation reaction; catalyst activation with benzyl alcohol; kinetic constants and rate equation for the *N*-alkylation reaction; isotopic effect kinetic study; characterization of the reused Hf-MOF-808_H_2_O (DRX, FESEM, TEM/EDX, TGA, ^13^C MAS NMR, and FTIR spectroscopies, elemental analysis, and GC-MS and NMR of the digested Hf-MOF-808_H_2_O sample); and liquid NMR spectra and data for the products obtained for the *N*-alkylation reactions (PDF)
