# Supplementary material for: Coordinatively Unsaturated Hf-MOF-808 Prepared via Hydrothermal Synthesis as a Bifunctional Catalyst for the Tandem N-Alkylation of Amines with Benzyl Alcohol
Source: ACS Sustain Chem Eng. 2021 Nov 17;9(47):15793–806. doi: 10.1021/acssuschemeng.1c04903 (PMC9153058; doi:10.1021/acssuschemeng.1c04903)
Supplement: Supplementary file 1 — sc1c04903_si_001.pdf [file sc1c04903_si_001.pdf]

Supporting Information

Coordinatively unsaturated Hf-MOF-808 prepared via hydrothermal synthesis as bifunctional catalyst for the tandem *N*-alkylation of amines with benzyl alcohol

Benjamin Bohigues,‡ Sergio Rojas-Buzo,‡ Manuel Moliner* and Avelino Corma*

Instituto de Tecnología Química, Universitat Politècnica de València - Consejo Superior de Investigaciones Científicas, Av. de los Naranjos, s/n, 46022 Valencia, Spain

*Corresponding authors: mmoliner@itq.upv.es, acorma@itq.upv.es

‡B. B. and S. R.-B. contributed equally to this work.

Number of pages: 28

Number of tables: 3

Number of figures: 11

Table S1. Solvent influence on the *N*-alkylation reaction of aniline **2a** and benzyl alcohol **1a**.

Entry	Solvent	3a (%)[c]	4a (%)[c]
**1**	DMF	0	< 1
**2**	DMSO	0	< 1
**3**	Butyl acetate	0	< 1
**4**	2-Methoxyethanol	0	< 1
**5**	1,2-Dichlorobenzene	3.2	39.5
**6**[b]	*o*-Xylene	0.9	85.3

[a] Reaction conditions: benzyl alcohol **1a** (0.60 mmol), aniline **2a** (0.60 mmol), Hf-MOF-808_H_2O (12 mol% Hf), solvent (1.35 mL), dodecane as external standard (0.22 mmol, 37.40 mg), T = 120°C, 3h. [b] 2h. [c] Yield determined by gas chromatography.

Table S2. Reaction steps, kinetic constants and rate equations for the *N*-alkylation reaction of aniline with benzyl alcohol.

Entry	Rate-determining step	Rate equation[a,b]
1	$PhCH_2OH + \text{Hf-MOF} \xrightarrow{k_a} PhCHO + \text{2H-Hf-MOF}$	$r_0=k_a[PhCH_2OH]$
2	$PhCHO + PhNH_2 \xrightarrow{k_b} PhCH{=}NPh + H_2O$	$r_0=k_b[PhCHO][PhNH_2]$
3	$PhCH{=}NPh + \text{2H-Hf-MOF} \xrightarrow{k_c} PhCH_2\text{-}NHPh + \text{Hf-MOF}$	$r_0=k_c[PhCH{=}NPh]=k_cK_b[PhCHO][PhNH_2]$

[a] k_a, k_b, and k_c are the kinetic constants for the three reaction steps. [b] According to the entire *N*-alkylation reaction and since the reaction mechanism consists of three steps (1), the steady-state approximation has been used with the imine intermediate as follow:

$$PhCH_2OH \xrightarrow{k_a} PhCHO + PhNH_2 \xrightarrow{k_b} PhCH{=}NPh \xrightarrow{k_c} PhCH_2NHPh \qquad (1)$$

Initially, the imine is generated from the condensation of aniline and benzaldehyde (Table S2, Entry 2) and hydrogenated to produce the *N*-benzylaniline (Table S2, Entry 3):

$$\frac{d[PhCH = NPh]}{dt} = 0 = k_b[PhCHO][PhNH_2] - k_c[PhCH = NPh]$$

$$[PhCH = NPh] = \frac{k_b}{k_c}[PhCHO][PhNH_2] = K_b[PhCHO][PhNH_2] \qquad (2)$$

K_b is the steady-state constant.

Finally, and using the rate equation in Table S2, Entry 3, the rate equation depends exclusively on the aldehyde and aniline concentration.

$$r_0 = k_c[PhCH = NPh] = k_cK_b[PhCHO][PhNH_2]$$

Table S3. Elemental analysis of the catalyst after the different uses.

Entry	MOF	C (%)	H (%)	N (%)
**1**	Hf-MOF-808_H_2O (4 uses)	23.9	2.9	1.16
**2**	Hf-MOF-808_H_2O fresh	15.4	3.9	0.0

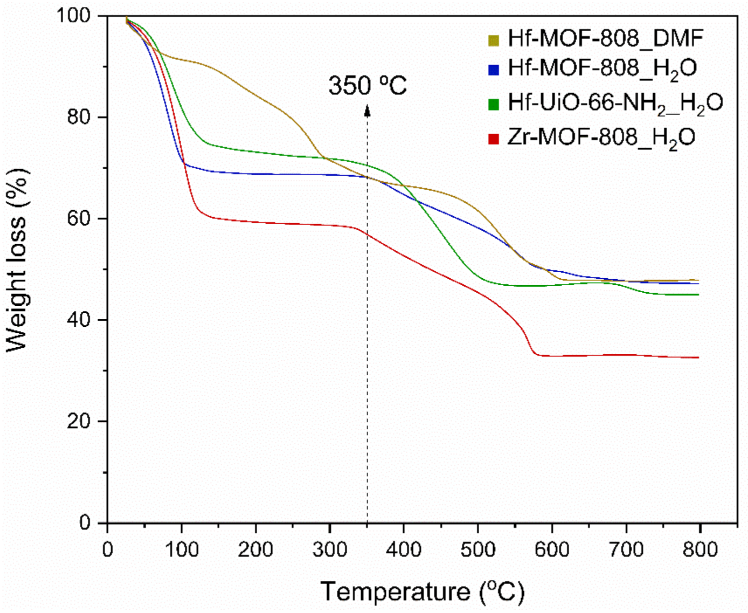

Figure S1. Thermogravimetric profiles (under aerobic atmosphere) of Hf- and Zr-MOFs. All these materials show thermal stability up to 350ºC.

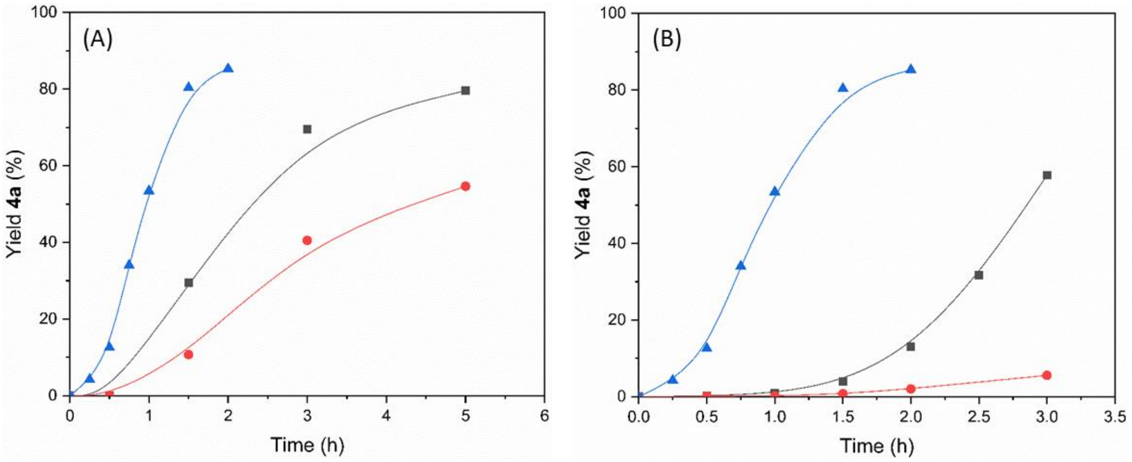

Figure S2. (A) *N*-alkylation reaction at 120ºC using Hf-MOF-808_H_2O as catalyst with different metal loadings: 12 mol% Hf (▲), 8 mol% Hf (■), and 5 mol% Hf (●). (B) *N*-alkylation reaction at 120 ºC (▲), 110 ºC (■), and 100ºC (●) using Hf-MOF-808_H_2O (12 mol% Hf) as catalyst. Reaction conditions: benzyl alcohol **1a** (0.60 mmol), aniline **2a** (0.60 mmol), Hf-MOF-808_H_2O as catalyst, solvent (1.35 mL), dodecane as external standard (0.22 mmol, 37.40 mg). Yield determined by gas chromatography.

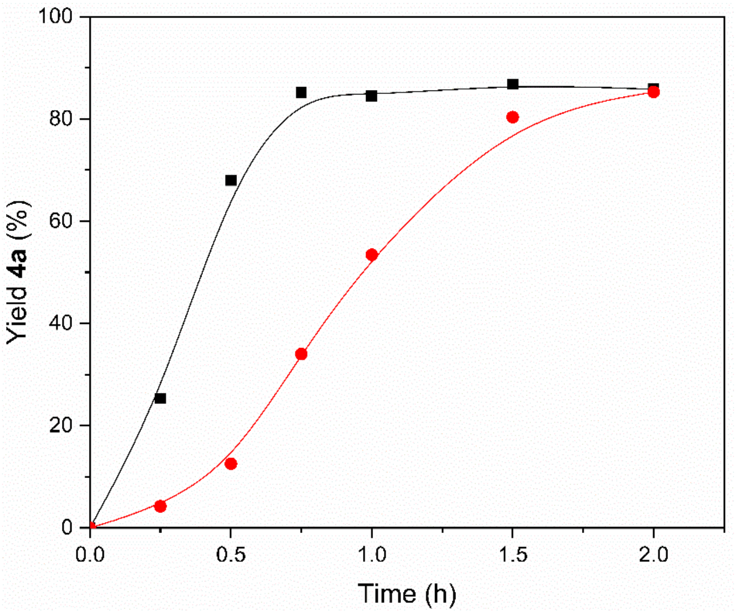

Figure S3. 4a yield obtained for the *N*-alkylation reaction of aniline with benzyl alcohol using (●) fresh Hf-MOF-808_H_2O and (■) Hf-MOF-808_H_2O previously activated at 120ºC for 2 h with benzyl alcohol. Reaction conditions: benzyl alcohol **1a** (0.60 mmol), aniline **2a** (0.60 mmol), Hf-MOF-808_H_2O (12 mol% Hf), solvent (1.35 mL), dodecane as external standard (0.22 mmol, 37.40 mg). Yield determined by gas chromatography.

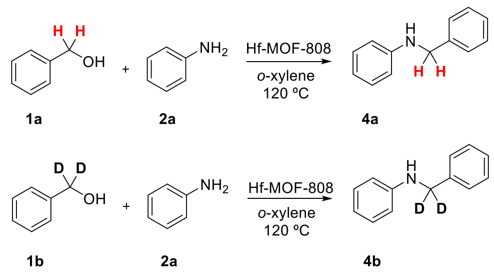

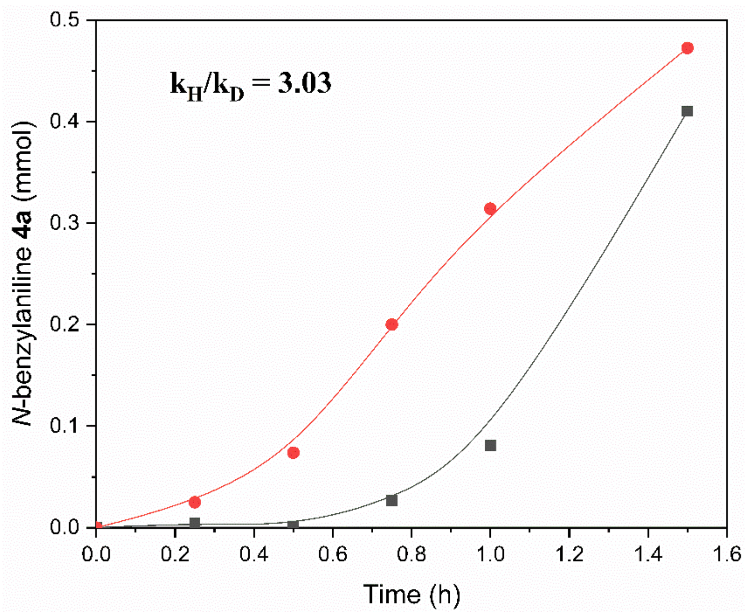

Figure S4. Kinetic study of the reaction of *N*-alkylation of benzyl alcohol **1a** (●) and benzyl alcohol-α, α-d_2 **1b** (■) (0.60 mmol in both cases) with aniline **2a** (0.60 mmol), Hf-MOF-808_H_2O (12 mol% metal), *o*-xylene (11.16 mmol, 1.35 mL), dodecane as external standard, T = 120°C. Conversion and performance determined by gas chromatography.

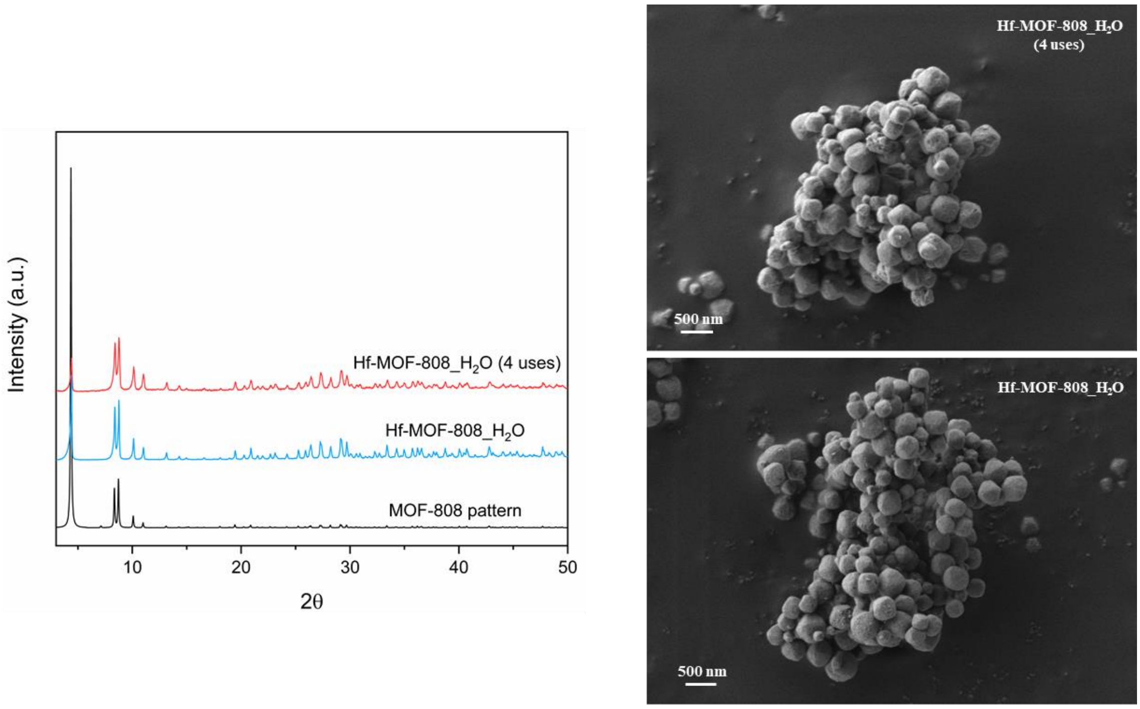

Figure S5. Characterization of Hf-MOF-808_H_2O recovered after the successive catalytic cycles for the *N*-alkylation reaction.

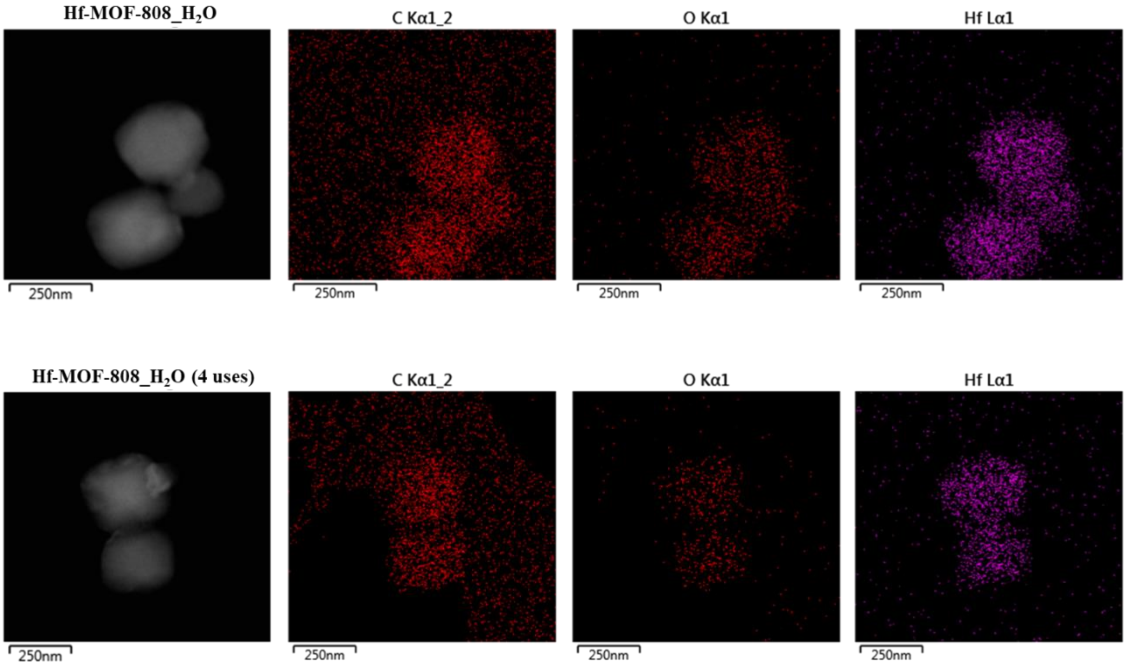

Figure S6. TEM/EDX analysis of fresh Hf-MOF-808_H_2O catalyst and Hf-MOF-808_H_2O after 4 re-uses.

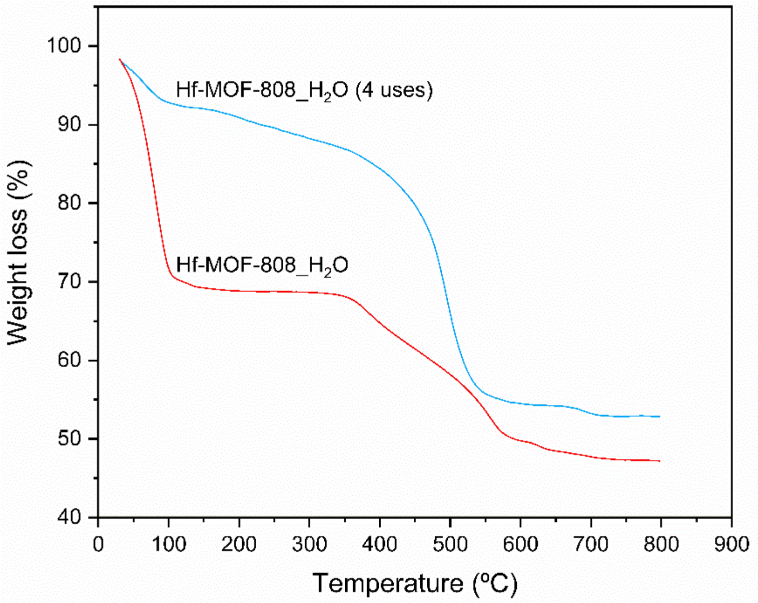

Figure S7. Thermogravimetric analysis of Hf-MOF-808_H_2O fresh and after 4 uses.

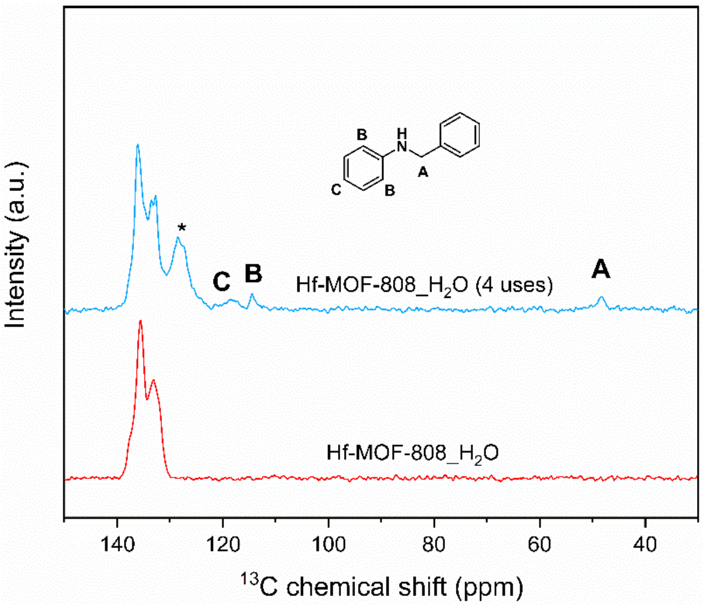

Figure S8. Solid-state ^{13}C MAS NMR spectra of fresh and reused Hf-MOF-808_H_2O (* residual signals of *o*-xylene).

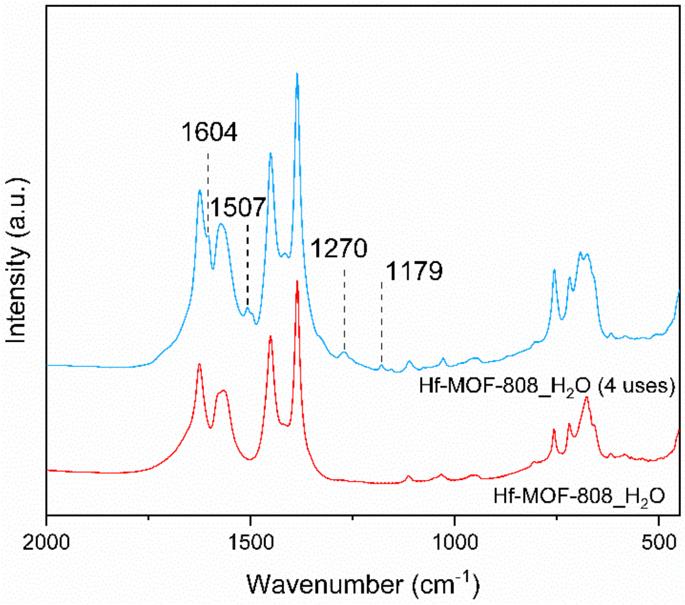

Figure S9. FT-IR analysis of the reused Hf-MOF-808_H_2O.

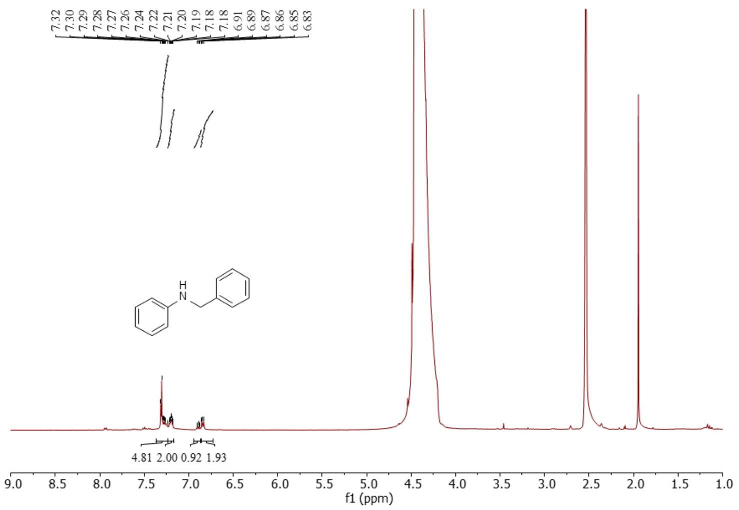

Figure S10. Liquid ^{1}H NMR spectrum of the reused Hf-MOF-808_H_2O digested with a D_2SO_4/DMSO-d_6 solution.

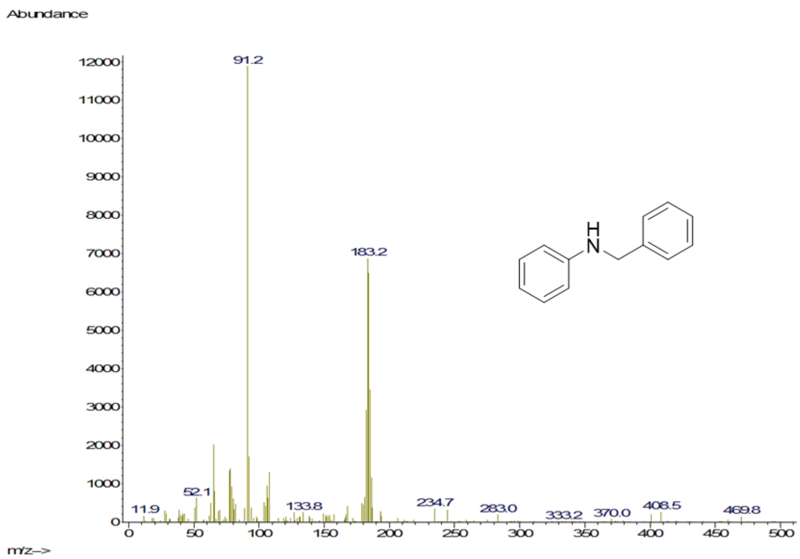

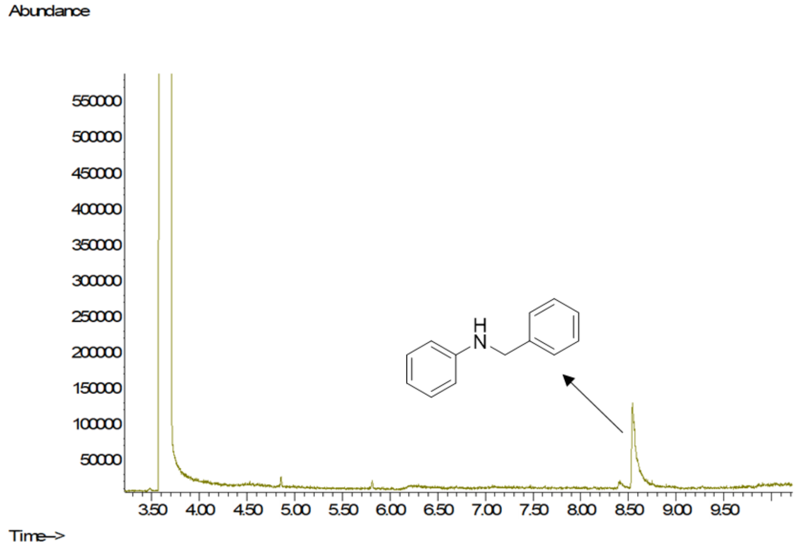

Figure S11. (Top) Fragmentation of the *N*-benzylaniline obtained after the disintegration of the reused Hf-MOF-808_H_2O and, (bottom) GC-MS chromatogram obtained from the solution after disintegrating the reused Hf-MOF-808_H_2O.

NMR data and spectra for products (**4**) obtained for the *N*-alkylation reaction

N-benzylaniline (**4a**).[1]

^{1}H NMR (300 MHz, $CDCl_3$) δ = 7.26-7.14 (m, 5H), 7.06 (t, 2H), 6.61 (t, 1H), 6.51 (d, 2H), 4.19 (s, 2H), 3.84 (br s, 1H). ^{13}C NMR (75 MHz, $CDCl_3$): δ =148.2, 139.6, 129.4, 128.7, 127.6, 127.3, 117.7, 112.9, 48.4.

N-(4-methylbenzyl)aniline (**4d**). (Table 4. Entry 1).[2]

^{1}H NMR (300 MHz, $CDCl_3$) δ = 7.14 (d, 2H), 7.08-7.03 (m, 4H), 6.60 (t, 1H), 6.51 (d, 2H), 4.15 (s, 2H), 3.81 (br s, 1H), 2.23 (s, 3H). ^{13}C NMR (75 MHz, $CDCl_3$): δ =148.3, 136.9, 136.5, 129.4, 129.3, 127.6, 117.6, 112.9, 48.2, 21.2.

N-(4-(methylthio)benzyl)aniline (**4f**). (Table 4. Entry 3).[2]

^{1}H NMR (300 MHz, $CDCl_3$) δ = 7.18 (dd, 4H), 7.09 (t, 2H), 6.64 (t, 1H), 6.54 (d, 2H), 4.20 (s, 2H), 3.89 (br s, 1H), 2.39 (s, 3H). ^{13}C NMR (75 MHz, $CDCl_3$): δ =148.1, 137.2, 136.4, 129.3, 128.1, 127.0, 117.7, 112.9, 47.9, 16.1.

N-(4-bromobenzyl)aniline (**4g**). (Table 4. Entry 4).[2]

^{1}H NMR (300 MHz, $CDCl_3$) δ = 7.38 (d, 2H), 7.23 (d, 2H), 7.08 (t, 2H), 6.64 (t, 1H), 6.52 (d, 2H), 4.25 (s, 2H), 3.99 (br s, 1H). ^{13}C NMR (75 MHz, $CDCl_3$): δ =147.9, 140.5, 132.4, 129.3, 127.3, 120.9, 117.8, 112.9, 48.0.

N-(4-methoxybenzyl)aniline (**4h**). (Table 4. Entry 5).[2]

^{1}H NMR (300 MHz, $CDCl_3$) δ = 7.21 (d, 2H), 7.09 (t, 2H), 6.86 (d, 2H), 6.63 (t, 1H), 6.55 (d, 2H), 4.17 (s, 2H), 3.86 (br s, 1H), 3.72 (s, 3H). ^{13}C NMR (75 MHz, $CDCl_3$): δ =158.9, 148.2, 131.4, 129.3, 128.8, 117.5, 114.1, 112.9, 55.3, 47.8.

N-butylaniline (**4i**). (Table 4. Entry 6).[3]

^{1}H NMR (300 MHz, $CDCl_3$) δ = 7.10 (t, 2H), 6.61 (t, 1H), 6.53 (d, 2H), 3.04 (t, 2H), 1.57-1.48 (m, 2H), 1.41-1.33 (m, 2H), 0.89 (t, 3H). ^{13}C NMR (75 MHz, $CDCl_3$): δ =148.6, 129.2, 117.1, 112.7, 43.7, 31.7, 20.3, 13.9.

N-benzyl-4-ethylaniline (**4k**). (Table 4. Entry 8).[4]

^{1}H NMR (300 MHz, $CDCl_3$) δ = 7.30-7.15 (m, 5H), 6.93 (d, 2H), 6.51 (d, 2H), 4.22 (s, 2H), 3.74 (br s, 1H), 2.49-2.43 (q, 2H), 1.11 (t, 3H). ^{13}C NMR (75 MHz, $CDCl_3$): δ =146.2, 139.7, 133.5, 128.6, 127.6, 127.2, 113.0, 48.7, 27.9, 15.9.

N-benzyl-4-bromoaniline (**4l**). (Table 4. Entry 9).[3]

^{1}H NMR (300 MHz, $CDCl_3$) δ = 7.25 (d, 4H), 7.22-7.17 (m, 1H), 7.16-7.12 (m, 2H), 6.40 (d, 2H), 4.19 (s, 2H), 3.96 (br s, 1H). ^{13}C NMR (75 MHz, $CDCl_3$): δ =147.1, 138.9, 132.0, 128.7, 127.4, 127.4, 114.5, 109.1, 48.2.

N-benzyl-3-nitroaniline (**4m**). (Table 4. Entry 10).[5]

^{1}H NMR (300 MHz, $CDCl_3$) δ = 7.44 (dd, 1H), 7.35 (t, 1H), 7.29-7.16 (m, 6H), 6.80 (dd, 1H), 4.30 (s, 2H). ^{13}C NMR (75 MHz, $CDCl_3$): δ =149.4, 148.8, 138.1, 129.8, 128.9, 127.7, 127.5, 118.7, 112.1, 106.6, 48.1.

N-benzyl-3-chloroaniline (**4n**). (Table 4. Entry 11).[6]

^{1}H NMR (300 MHz, $CDCl_3$) δ = 7.28-7.17 (m, 5H), 6.96 (t, 1H), 6.58 (d, 1H), 6.51 (t, 1H), 6.38 (d, 1H), 4.20 (s, 2H), 4.00 (br s, 1H). ^{13}C NMR (75 MHz, $CDCl_3$): δ =149.3, 138.8, 135.1, 130.2, 128.8, 127.5, 127.4, 117.5, 112.6, 111.2, 48.1.

N-benzylaniline (**4a**).

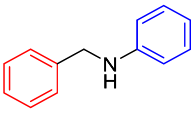

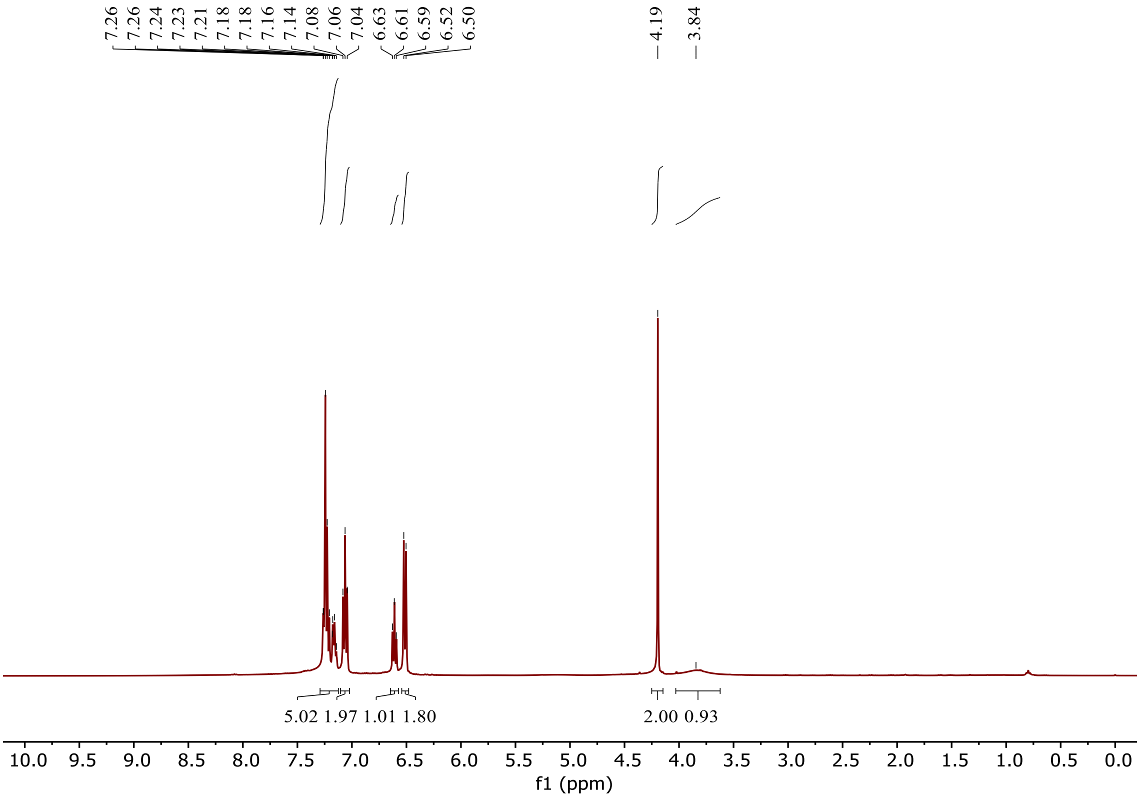

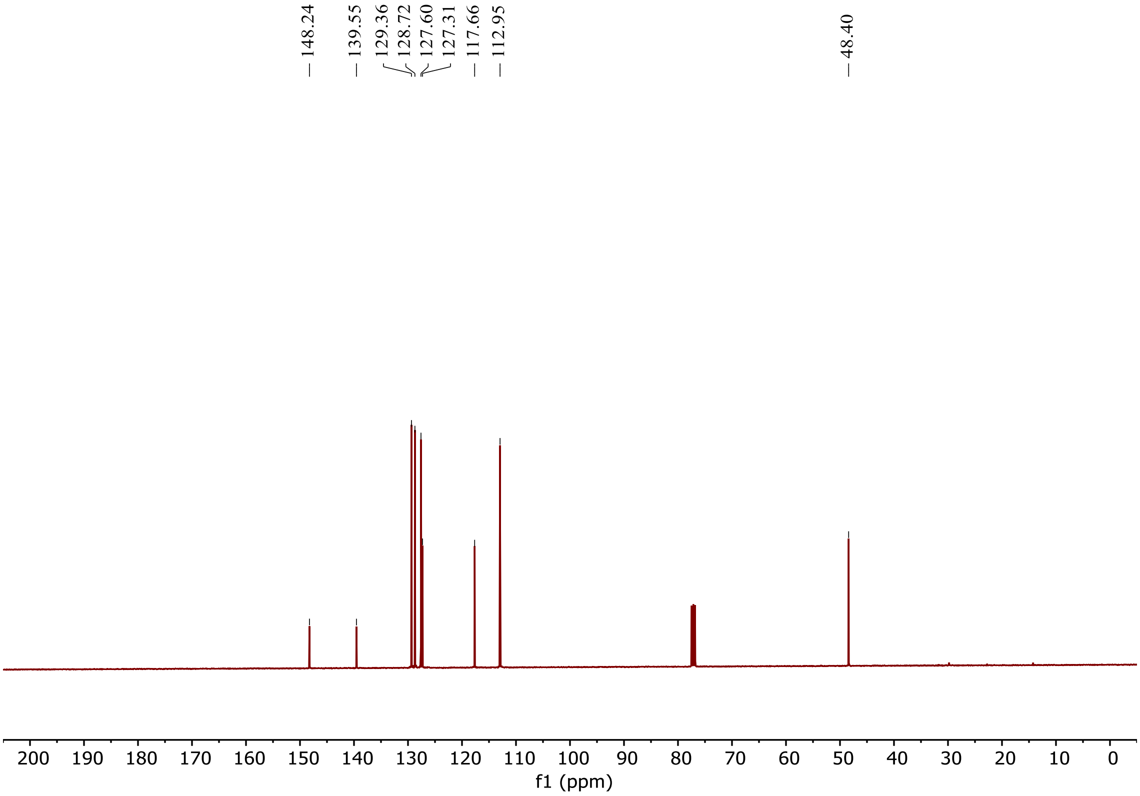

N-(4-methylbenzyl)aniline (**4d**).

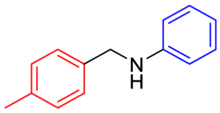

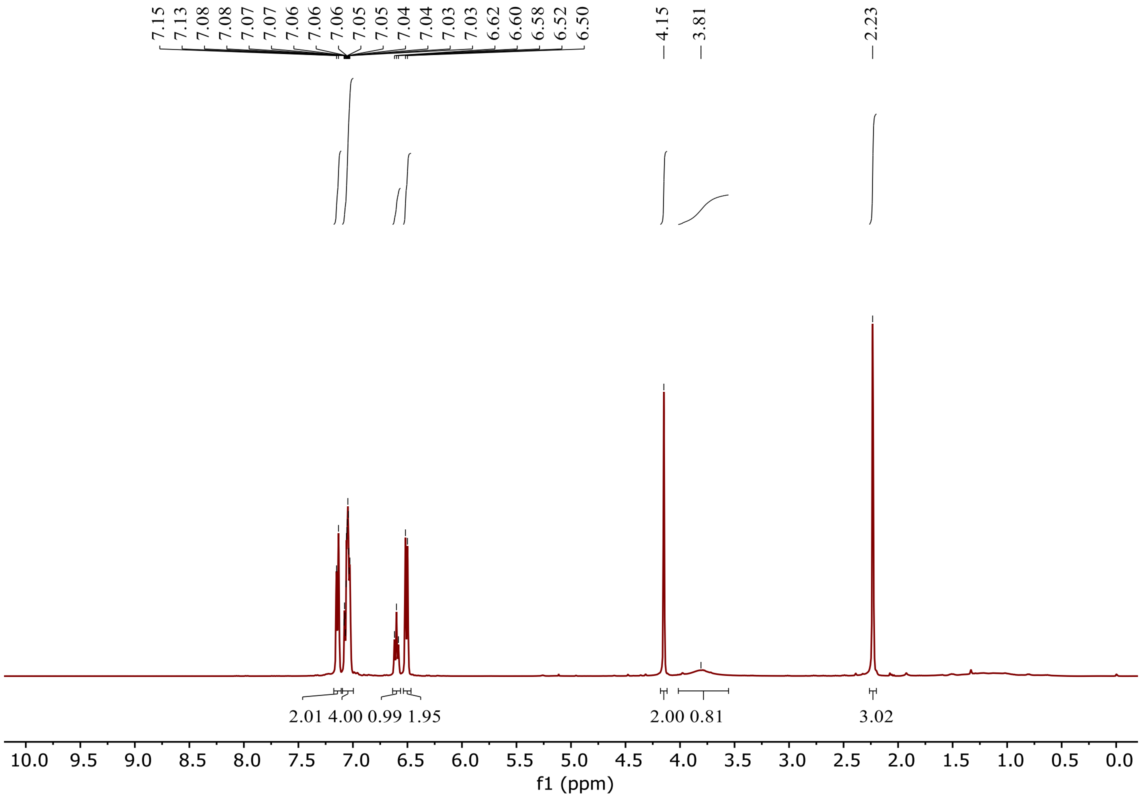

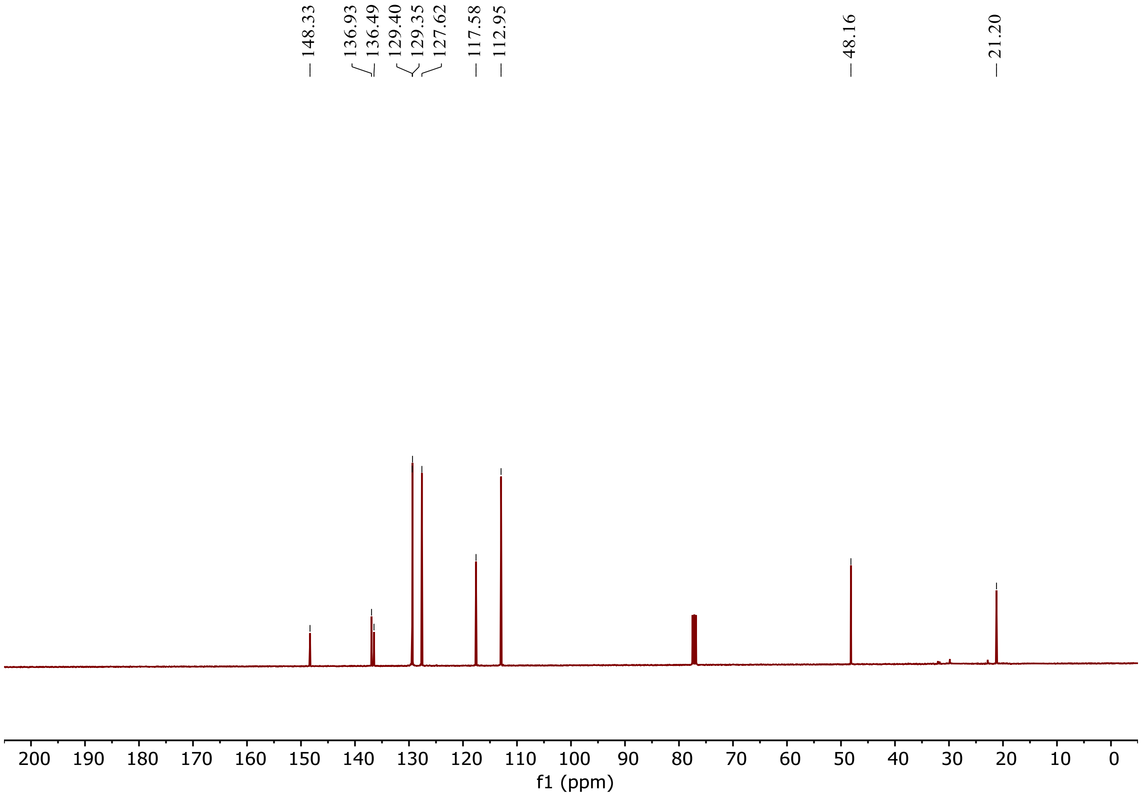

N-(4-(methylthio)benzyl)aniline (**4f**).

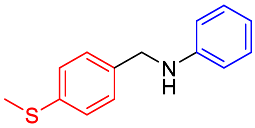

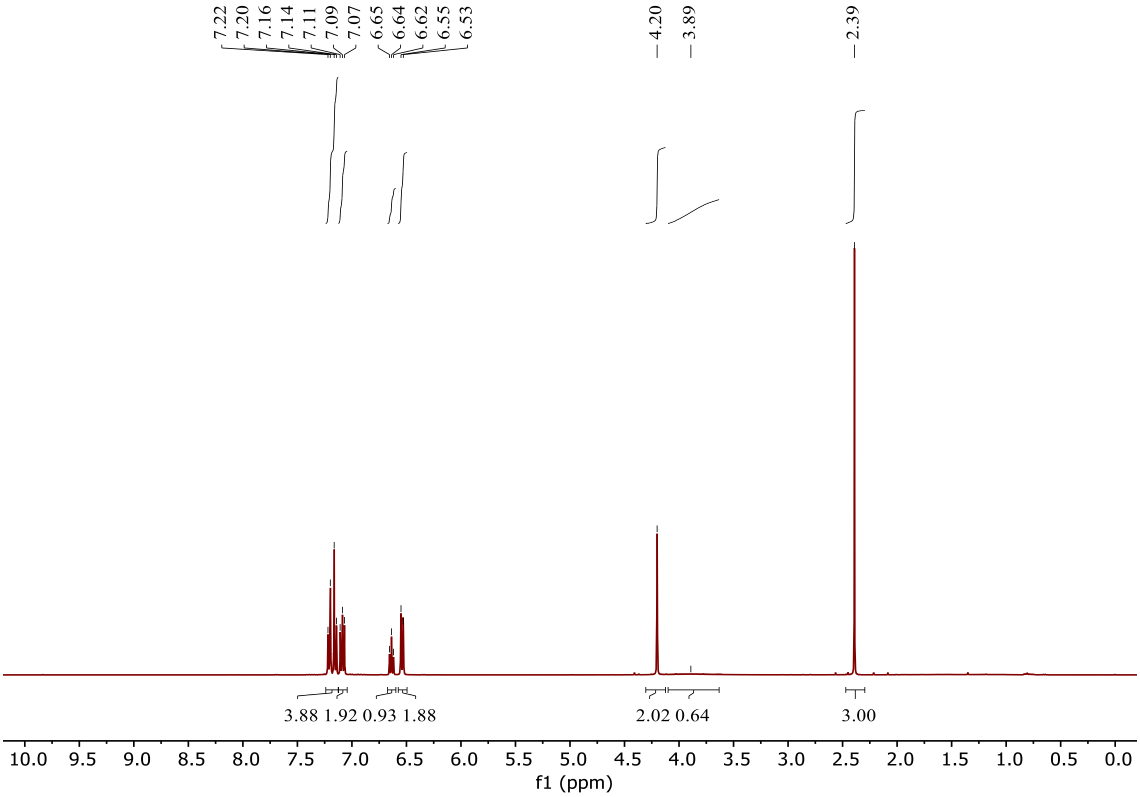

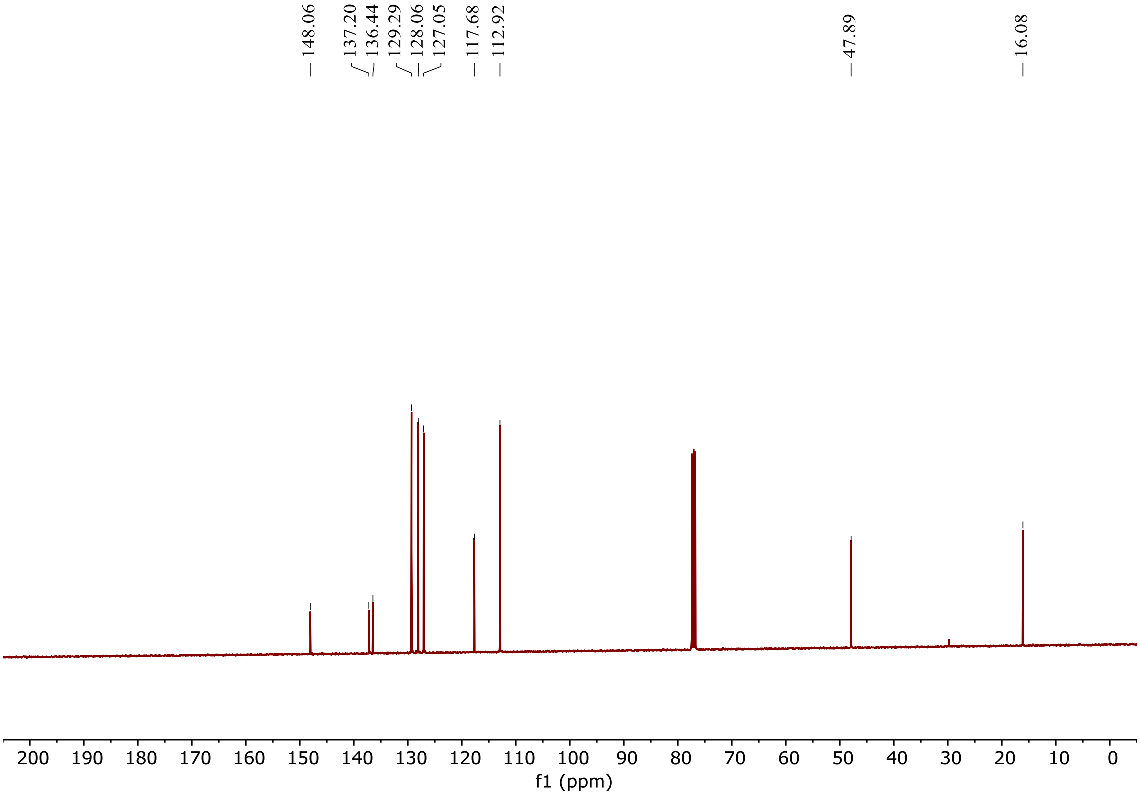

N-(4-bromobenzyl)aniline (**4g**).

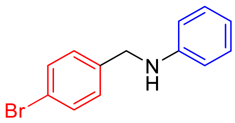

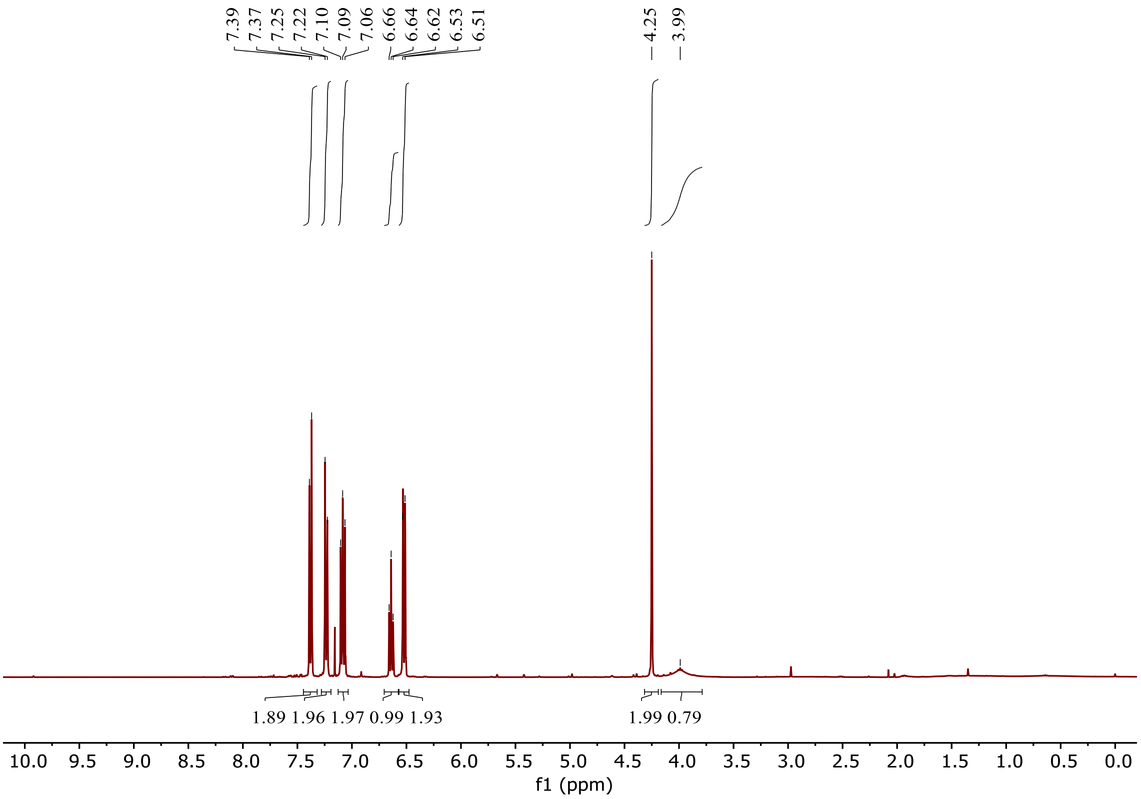

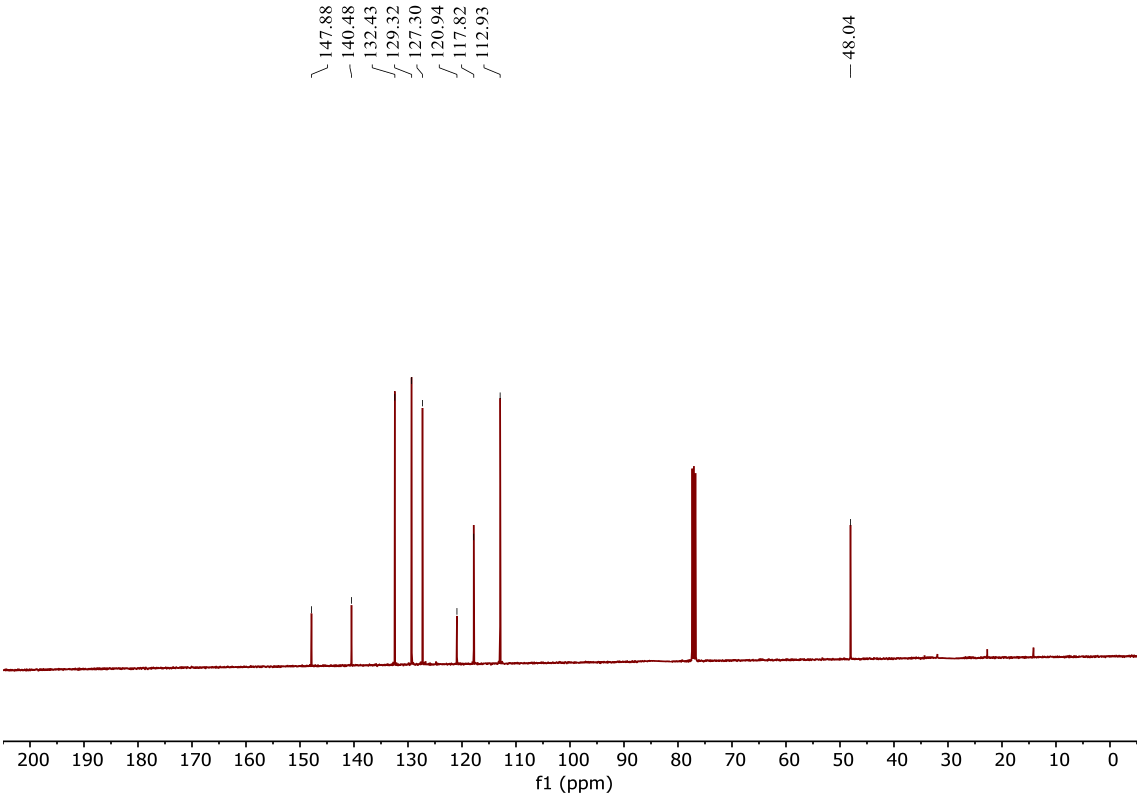

N-(4-methoxybenzyl)aniline (**4h**).

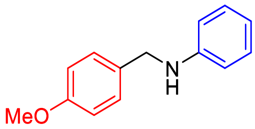

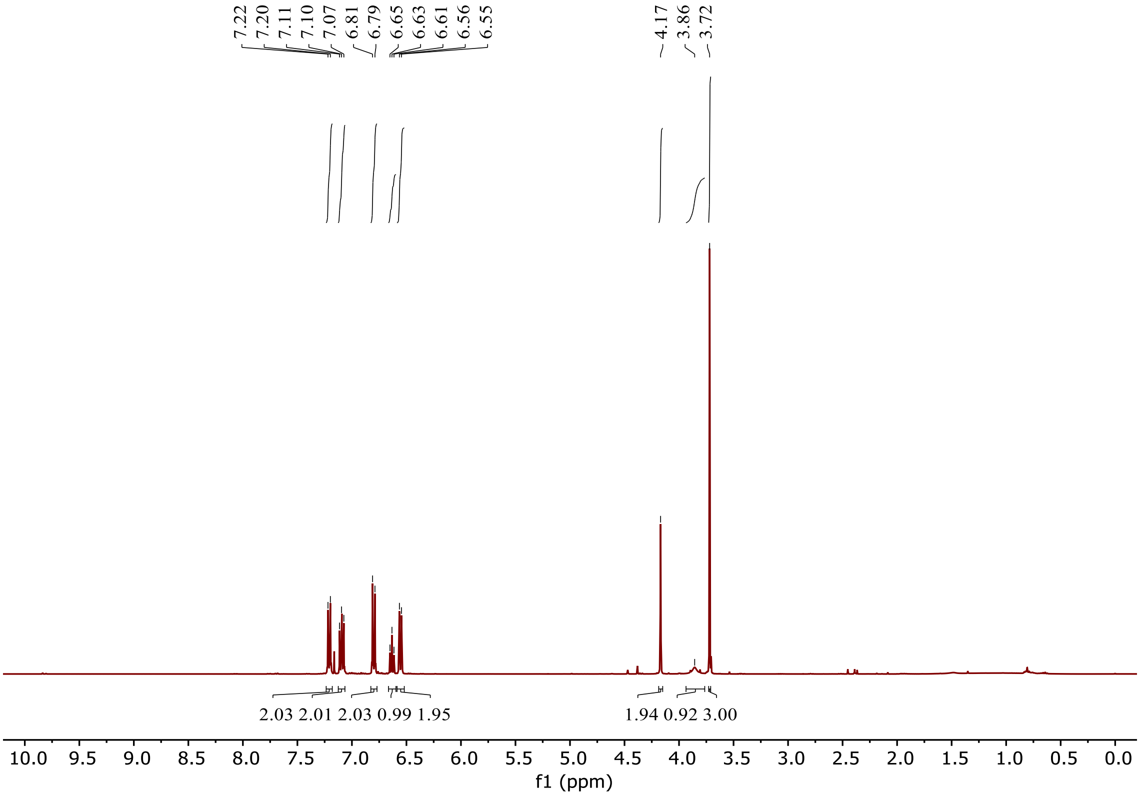

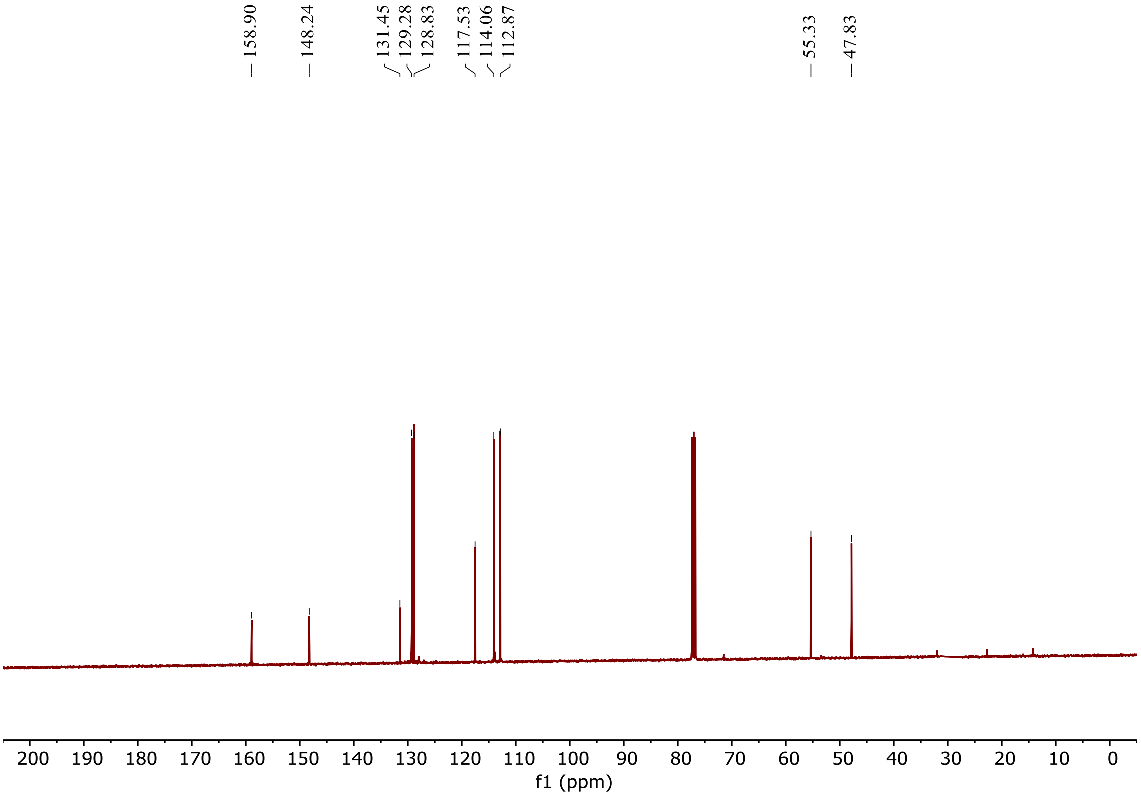

N-Butylaniline (**4i**).

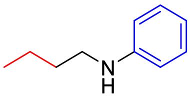

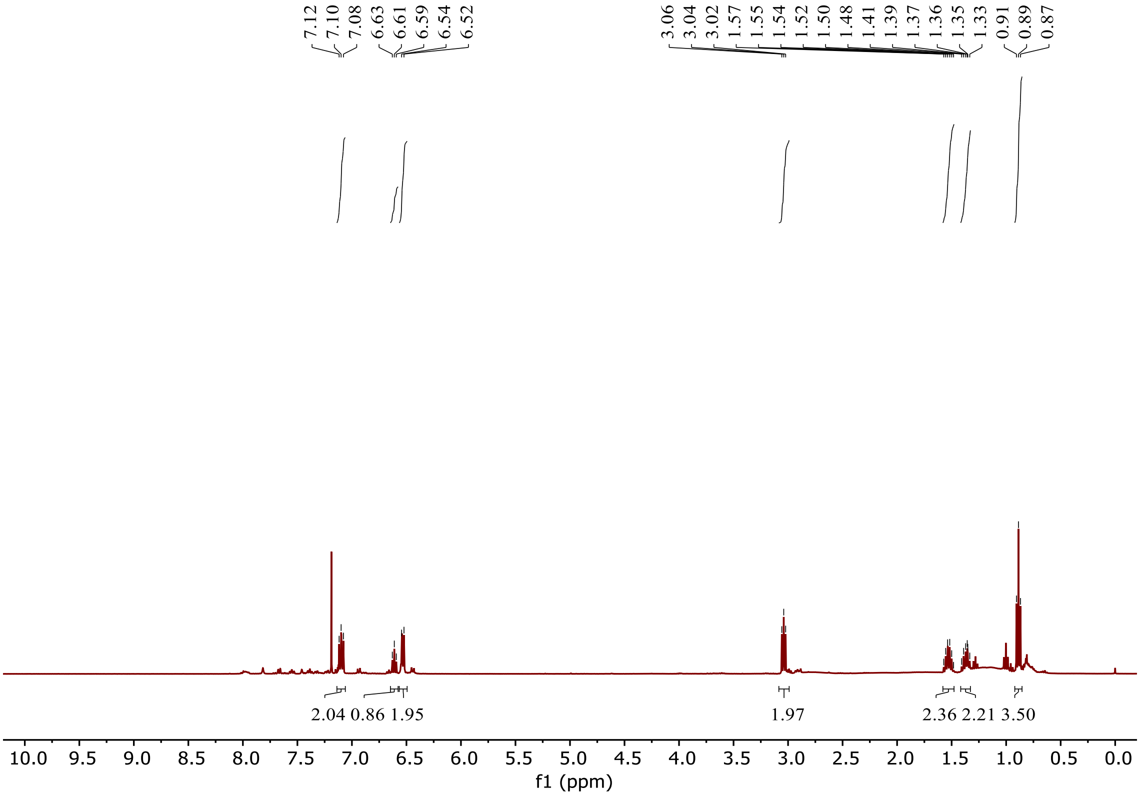

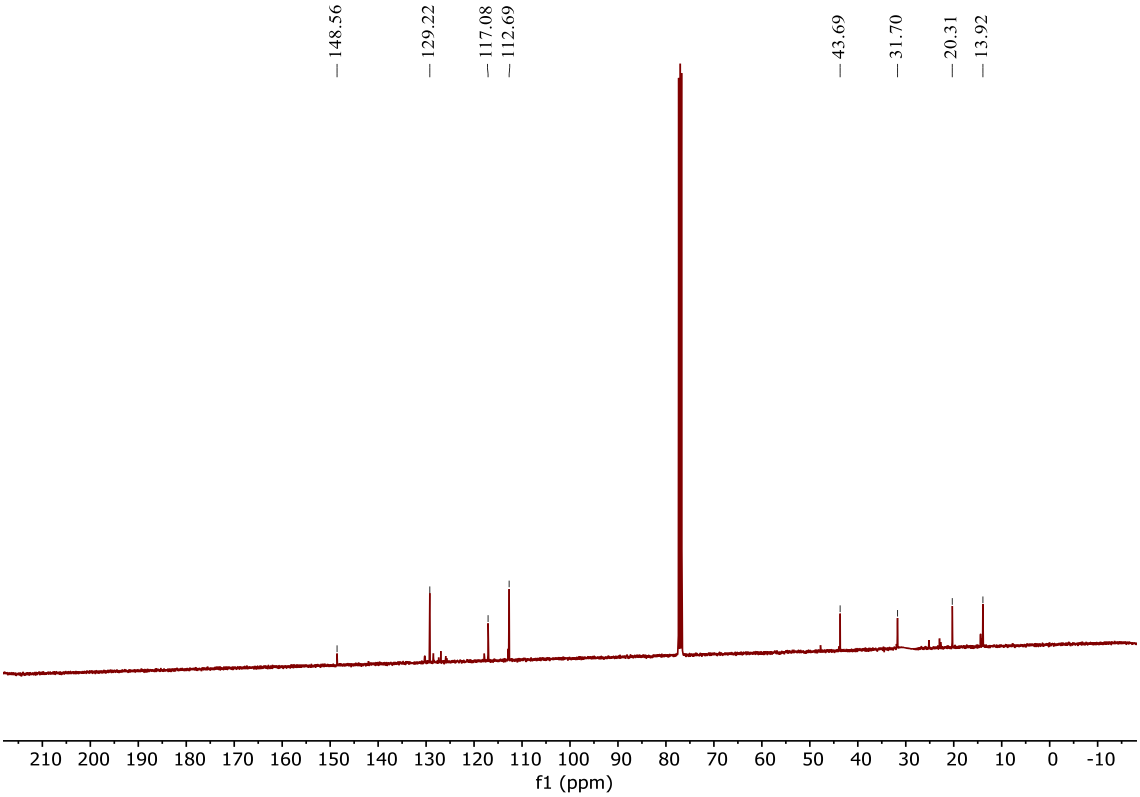

N-benzyl-4-ethylaniline (**4k**).

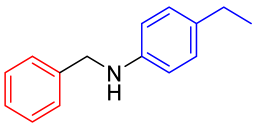

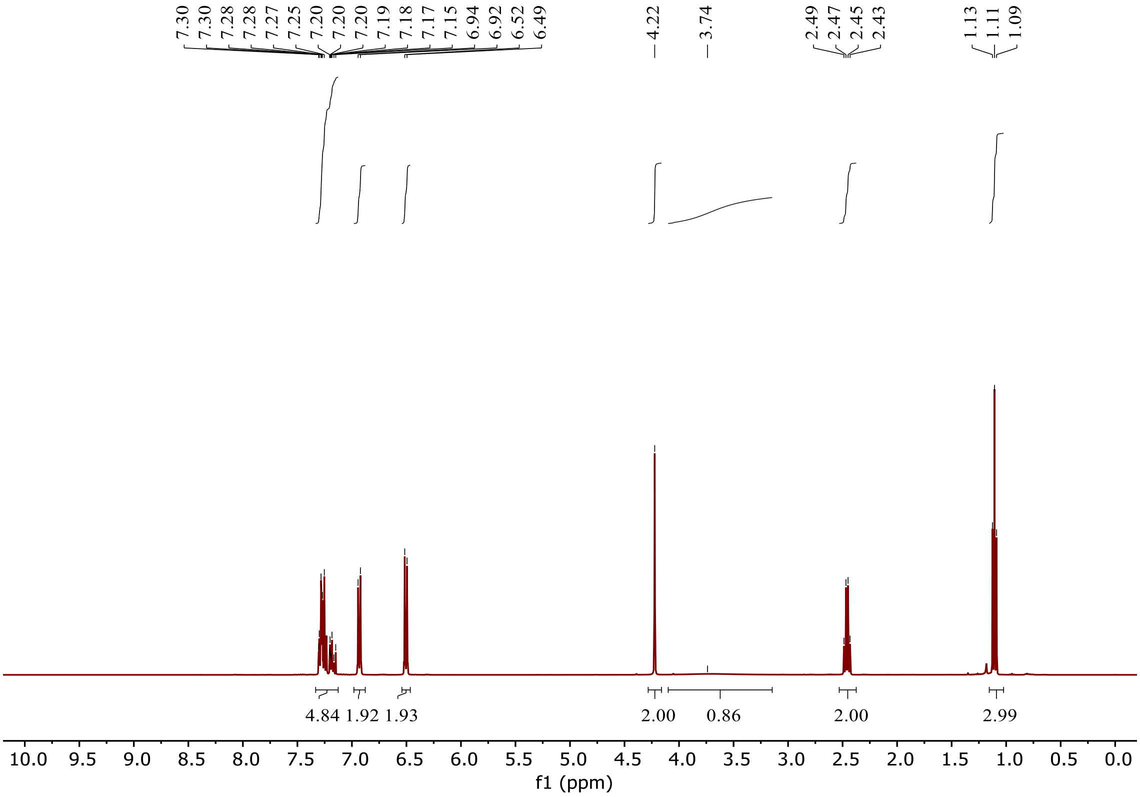

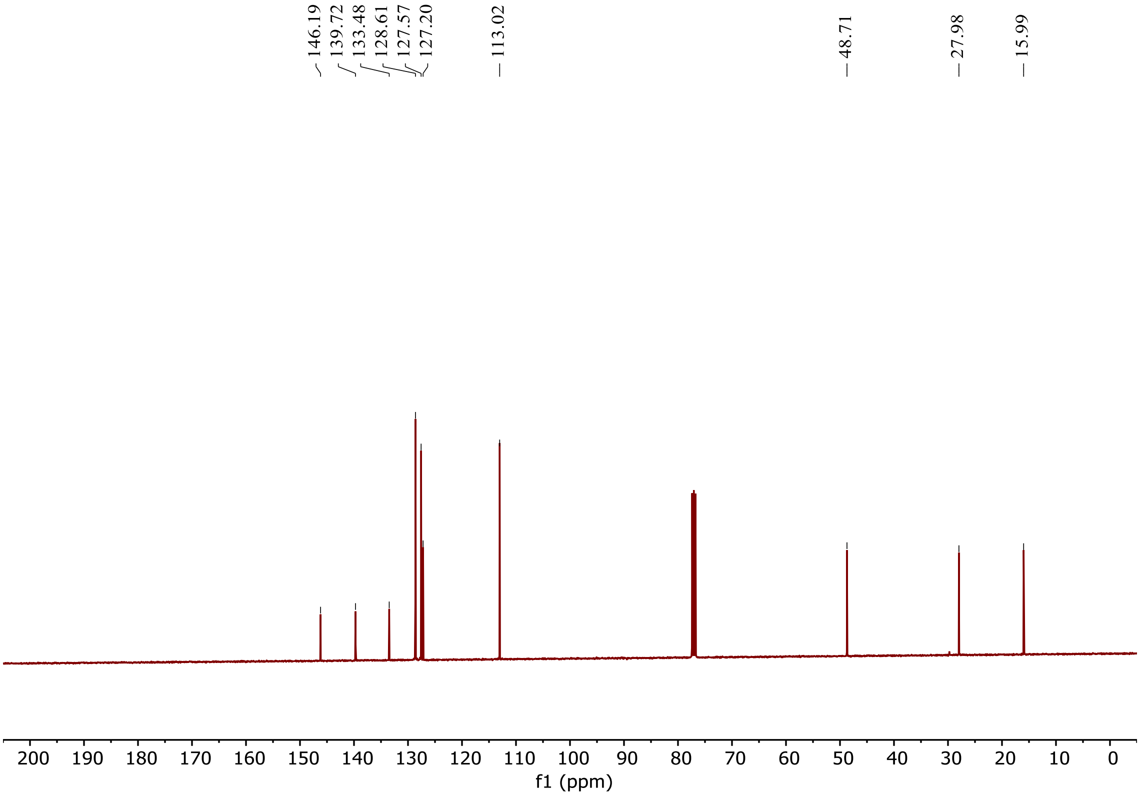

N-benzyl-4-bromoaniline (**4l**).

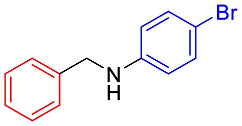

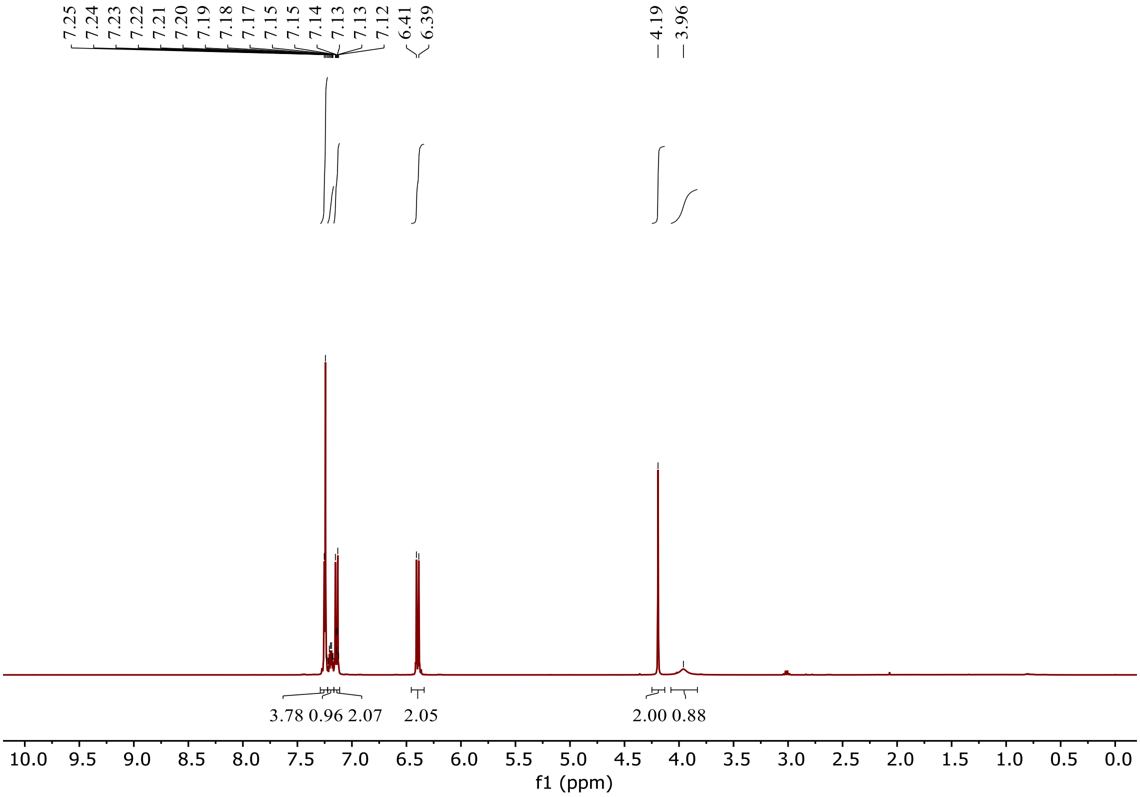

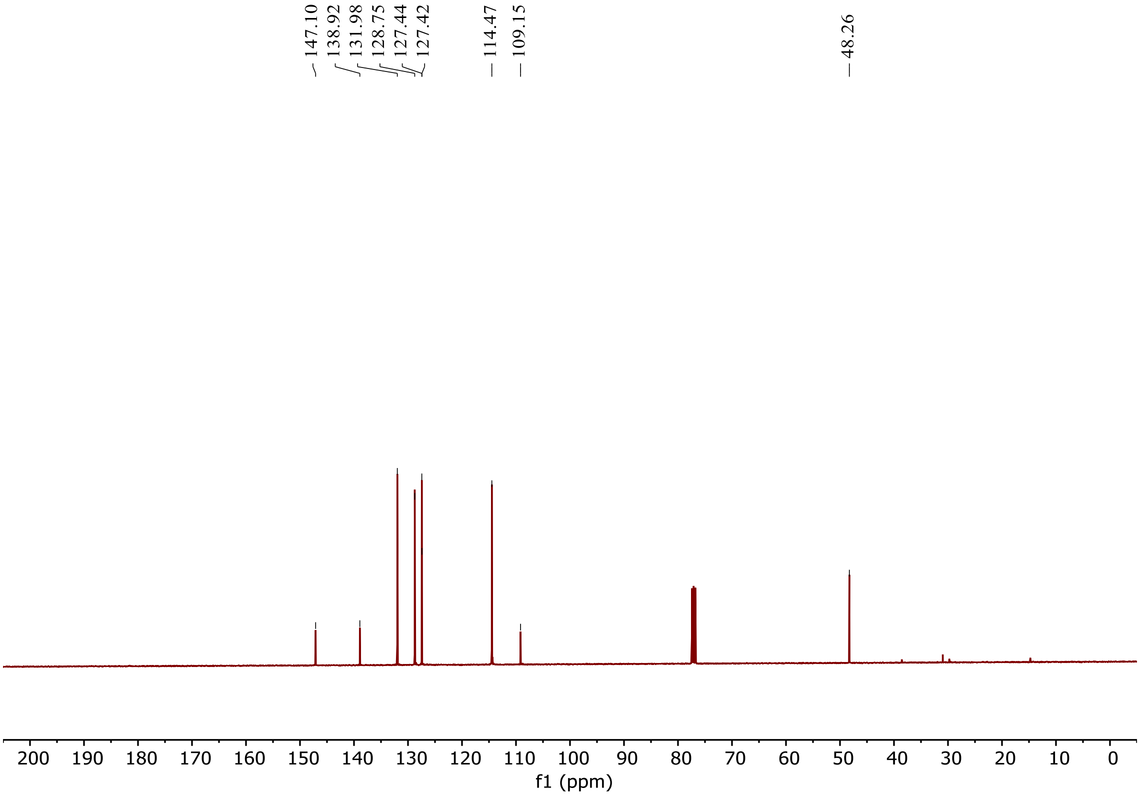

N-benzyl-3-nitroaniline (**4m**).

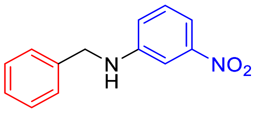

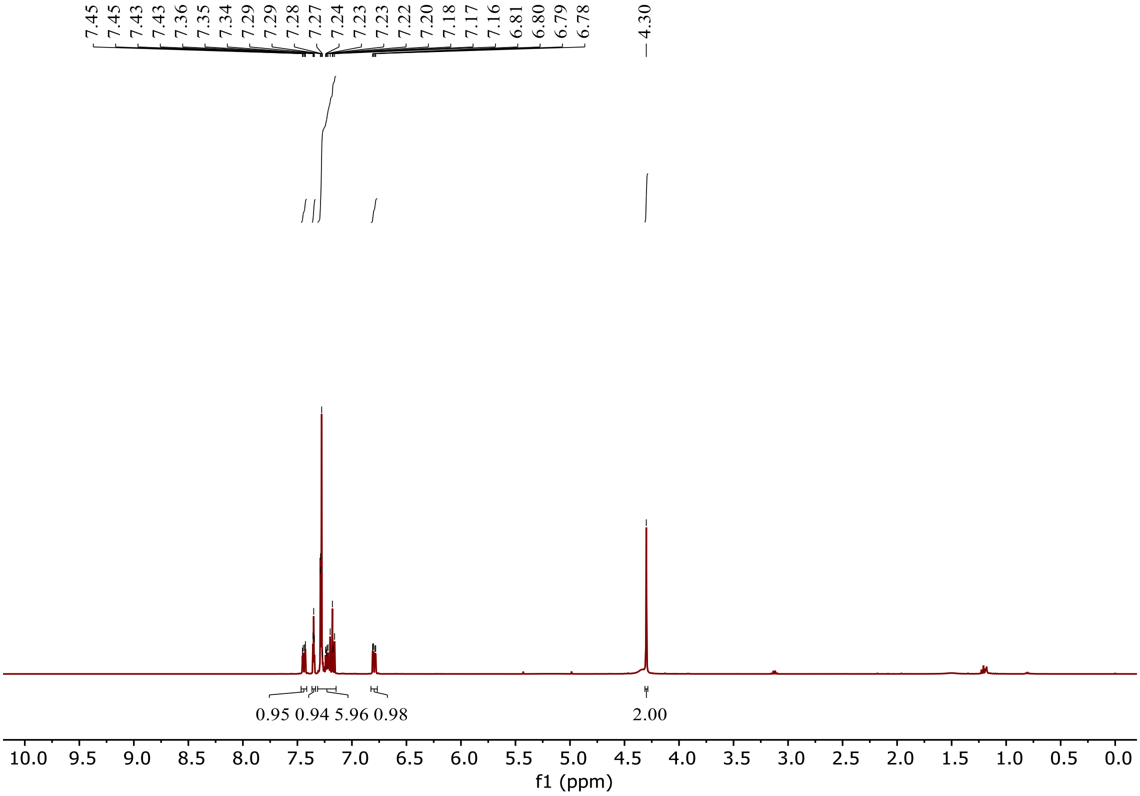

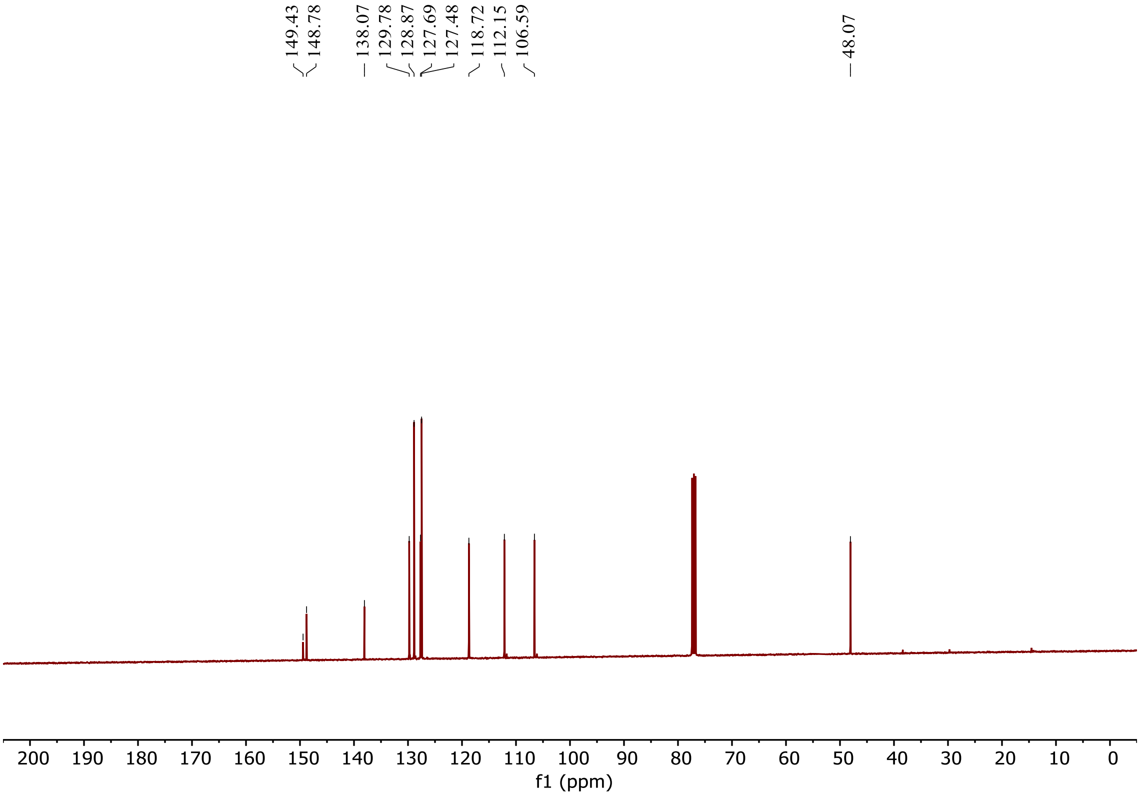

N-benzyl-3-chloroaniline (**4n**).

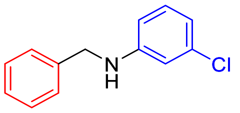

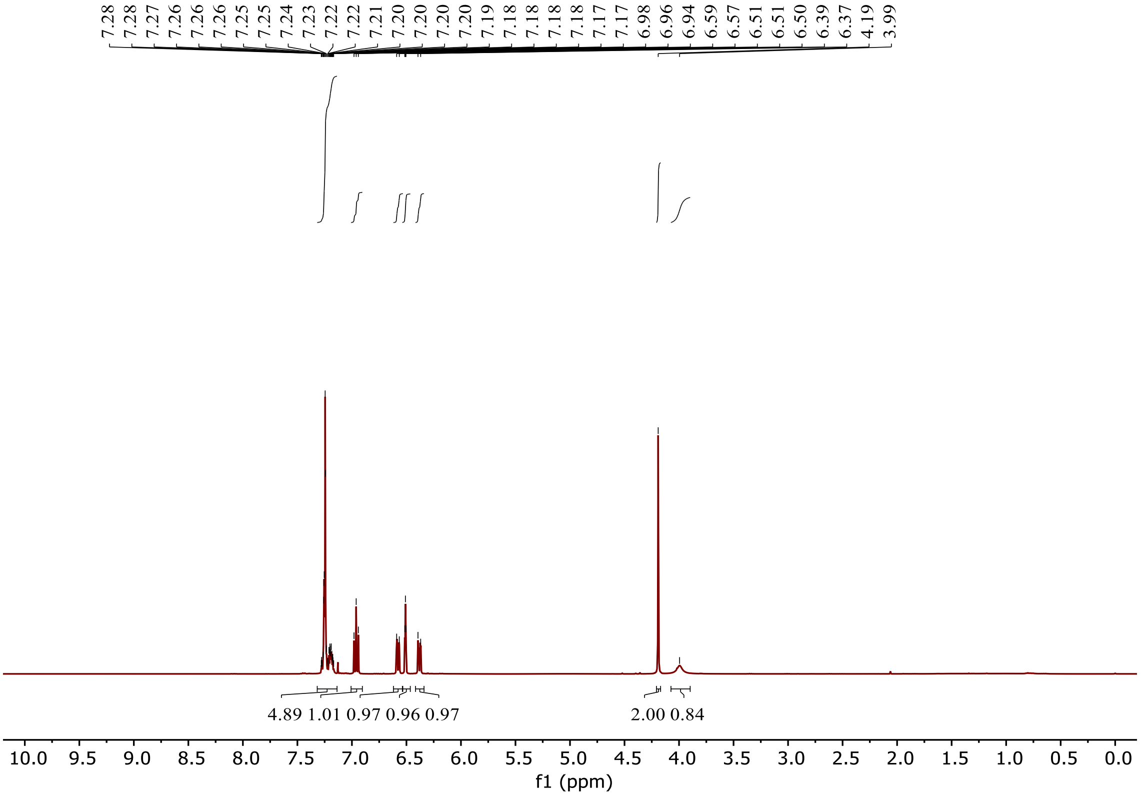

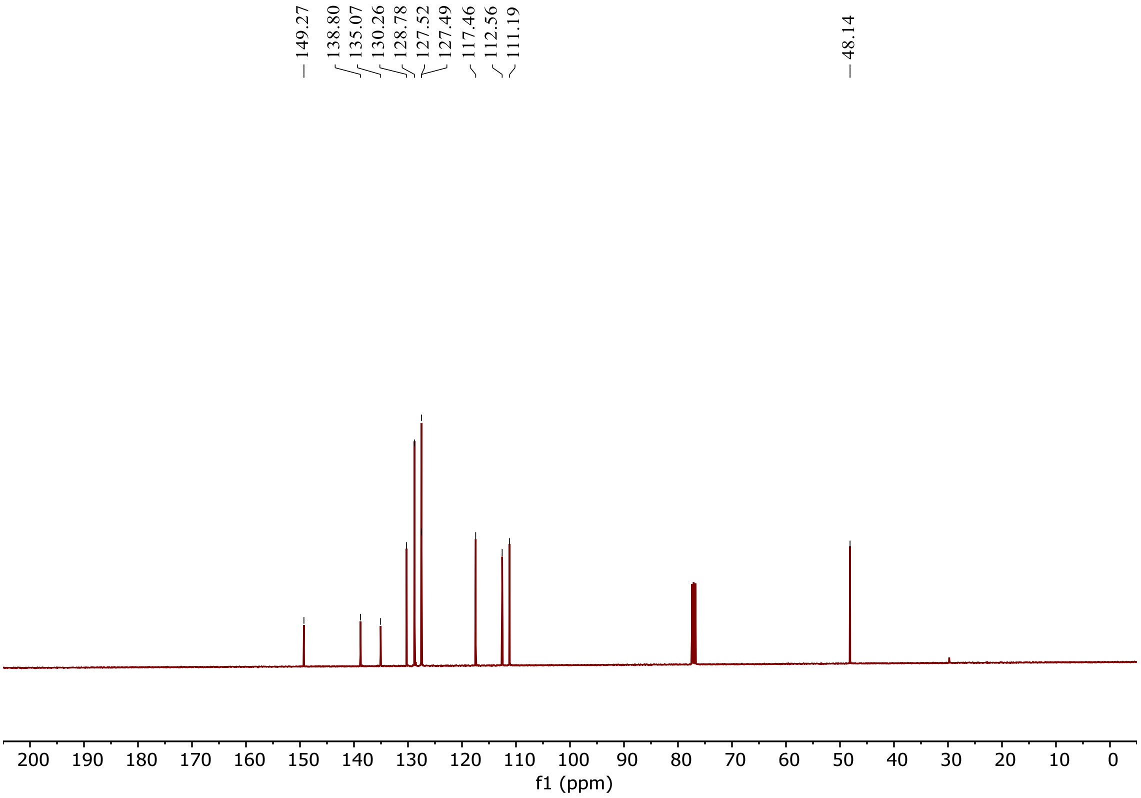

REFERENCES

(1) Gutiérrez-Tarriño, S.; Rojas-Buzo, S.; Lopes, C. W.; Agostini, G.; Calvino, J. J.; Corma, A.; Oña-Burgos, P. Cobalt Nanoclusters Coated with N-Doped Carbon for Chemoselective Nitroarene Hydrogenation and Tandem Reactions in Water. *Green Chem.* **2021**, *23* (12), 4490–4501. https://doi.org/10.1039/D1GC00706H.

(2) Li, W.; Huang, M.; Liu, J.; Huang, Y.-L.; Lan, X.-B.; Ye, Z.; Zhao, C.; Liu, Y.; Ke, Z. Enhanced Hydride Donation Achieved Molybdenum Catalyzed Direct N -Alkylation of Anilines or Nitroarenes with Alcohols: From Computational Design to Experiment. *ACS Catal.* **2021**, *11* (16), 10377–10382. https://doi.org/10.1021/acscatal.1c02956.

(3) Vellakkaran, M.; Singh, K.; Banerjee, D. An Efficient and Selective Nickel-Catalyzed Direct N-Alkylation of Anilines with Alcohols. *ACS Catal.* **2017**, *7* (12), 8152–8158. https://doi.org/10.1021/acscatal.7b02817.

(4) Fertig, R.; Irrgang, T.; Freitag, F.; Zander, J.; Kempe, R. Manganese-Catalyzed and Base-Switchable Synthesis of Amines or Imines via Borrowing Hydrogen or Dehydrogenative Condensation. *ACS Catal.* **2018**, *8* (9), 8525–8530. https://doi.org/10.1021/acscatal.8b02530.

(5) Reddy, M. M.; Kumar, M. A.; Swamy, P.; Naresh, M.; Srujana, K.; Satyanarayana, L.; Venugopal, A.; Narender, N. N-Alkylation of Amines with Alcohols over Nanosized Zeolite Beta. *Green Chem.* **2013**, *15* (12), 3474. https://doi.org/10.1039/c3gc41345d.

(6) Xu, Z.; Yu, X.; Sang, X.; Wang, D. BINAP-Copper Supported by Hydrotalcite as an Efficient Catalyst for the Borrowing Hydrogen Reaction and Dehydrogenation Cyclization under Water or Solvent-Free Conditions. *Green Chem.* **2018**, *20* (11), 2571–2577. https://doi.org/10.1039/C8GC00557E.
